# Therapeutic approaches for septicemia induced by multidrug-resistant bacteria using desert-adapted plants

**DOI:** 10.3389/fcimb.2025.1493769

**Published:** 2025-04-22

**Authors:** Nesreen Safwat, Rana Elshimy, Soha O. Hassanin, Arwa Ramadan El-manakhly, Abdullah N. Noaf, Abdallah Tageldein Mansour, Fatma Alshehri, Majid Alhomrani, Abdulhakeem S. Alamri, Mahmoud Mohammed Bendary

**Affiliations:** ^1^ Department of Microbiology and Immunology, Faculty of pharmacy, Modern University for Technology and Information, Cairo, Egypt; ^2^ Department of Microbiology and Immunology, Faculty of Pharmacy, Alhram Canadian University, Giza, Egypt; ^3^ Department of Biochemistry, Faculty of pharmacy, Modern University for Technology and Information, Cairo, Egypt; ^4^ Surgical Department, Al Hadithah General Hospital, Al-Qurayyat, Saudi Arabia; ^5^ Fish and Animal Production Department, College of Agriculture and Food Sciences, King Faisal University, Al-Ahsa, Saudi Arabia; ^6^ Department of Biology, College of Sciences, Princess Nourah bint Abdulrahman University, Riyadh, Saudi Arabia; ^7^ Department of Clinical Laboratories Sciences, The Faculty of Applied Medical Science, Taif University, Taif, Saudi Arabia; ^8^ Research Center for health science, Deanship of Scientific Research, Taif University, Taif, Saudi Arabia; ^9^ Department of Microbiology and Immunology, Faculty of Pharmacy, Port Said University, Port Said, Egypt

**Keywords:** septic patients, gram-negative, medicinal plants, *J. candicans*, amikacin

## Abstract

**Aim:**

Septicemia, a life-threatening condition, can arise when bacterial infections are left untreated, allowing the pathogens to spread into the bloodstream. Moreover, infections caused by MDR bacteria are particularly challenging, as they can persist and lead to septicemia even when treated with conventional antibiotics. This study aimed to address this crisis by investigating combination therapies using desert-adapted medicinal plant extracts, including *Jasonia candicans (J. candicans)*, *Cistanche tubulosa*, *Moltkiopsis ciliata*, and *Thymelea hirsuta*, as alternative treatments. The goal was to develop new strategies to combat resistance and improve the management of septic patients.

**Methodology:**

In this study, 400 blood samples from septic patients were analyzed to identify Gram-negative bacterial isolates. Antimicrobial resistance patterns were assessed using standard susceptibility tests. Medicinal plant extracts were evaluated for antimicrobial activity using agar diffusion and broth microdilution assays, while COX-1 and COX-2 inhibition and antioxidant activity were measured using *in vitro* assays. Histopathological examinations were conducted on treated mice to assess tissue damage and response.

**Results:**

We observed a high prevalence of *E. coli* and *K. pneumoniae* among septic patients. Multidrug resistance was widespread, with many isolates showing high resistance to various antibiotics, although all were susceptible to colistin. Evaluation of desert-adapted plant extracts revealed that *J. candicans* exhibited the most potent antimicrobial activity and the strongest COX-1 and COX-2 inhibitory activities, as well as antioxidant effects, compared to other extracts and Celecoxib, with a concentration required to achieve 50% enzyme inhibition (IC_50_) value of 71.97 μg/mL for antioxidant activity. Moreover, the combination of this extract with amikacin showed a synergistic effect, significantly enhancing antimicrobial efficacy and converting over 50% of amikacin-resistant strains to sensitive phenotypes. Histopathological analysis of mice showed that the combination of *J. candicans* extract and amikacin resulted in reduced severity of pulmonary lesions and splenic damage compared to amikacin alone.

**Conclusion:**

We highlighted the potential of *J. candicans* extracts as combination therapies alongside traditional antibiotics for combating MDR Gram-negative infections, due to their superior antimicrobial, anti-inflammatory, and antioxidant properties.

## Introduction

Septicemia is a serious and potentially fatal condition that occurs when bacteria enter the bloodstream and spread throughout the body, triggering a widespread immune response. This condition can develop from infections caused by various bacterial species, but it is especially concerned with resistant strains, such as multidrug-resistant (MDR) bacteria. These resistant pathogens pose a significant challenge to treatment because they have developed the ability to withstand many commonly used antibiotics, making infections more difficult to control and eradicate. Even with timely medical intervention, MDR infections can still progress to septicemia due to the limited effectiveness of existing antimicrobial therapies (Tang et al., 2024). Infections with MDR- Gram-negative bacteria can trigger an uncontrolled inflammatory response that impairs organ function, resulting in a complex syndrome characterized by oxidative stress, immune dysfunction, impaired tissue oxygenation, and hypercoagulability (Prescott and Angus, 2018; American Thoracic Society, 2018). When microorganisms invade the body, the host’s immune system attempts to clear these pathogens through an inflammatory response, primarily mediated by the activation of neutrophils and macrophages/monocytes. This response, however, can become excessive and uncontrolled, leading to irreversible tissue damage and potentially death (Singer et al., 2016; Tang et al., 2024). The balance between inflammation and the immune response is critical in the context of infections. While inflammation is a necessary component of the body’s defense mechanism against pathogens, an uncontrolled inflammatory response can lead to tissue damage and contribute to the development of sepsis, especially in the presence of MDR bacteria. Cyclooxygenase-2 (COX-2) inhibition can shift this balance by modulating the inflammatory response, potentially allowing for a more targeted and effective immune response against the infection. Furthermore, reducing inflammation may minimize the risk of systemic inflammatory responses, which can lead to severe complications. This reduction in inflammation can potentially improve tissue healing and recovery, enhance blood flow to the affected area, and promote a more effective immune response. A more balanced immune response is crucial when managing infections, particularly those caused by MDR pathogens, as these bacteria often exploit the inflammatory environment to thrive and evade immune detection (Medzhitov, 2008).

Of note, the broad-spectrum antibiotics commonly used for empirical treatment of sepsis are associated with adverse outcomes, including the disruption of normal microbiota and the selection for resistant pathogens (Busani et al., 2019; Zilberman-Itskovich and Goldberg, 2022). The rise of MDR- Gram-negative bacteria such as *Klebsiella pneumoniae (K. pneumoniae)*, *Pseudomonas aeruginosa (P. aeruginosa)*, and *Acinetobacter baumannii (A. baumannii)*, which are resistant to carbapenems, the last line of defense, underscores the urgent need for new therapeutic strategies (Pop-Vicas and Opal, 2013). Current sepsis management, which includes supportive care such as organ function support, intravenous fluids, antibiotics, and oxygen, remains largely nonspecific and has limitations. Considering the critical role of inflammation in sepsis, innovative therapies targeting the host immune response could improve outcomes (Liang et al., 2015). Of note, understanding the mechanisms of resistance in these pathogens can facilitate the identification and development of novel antimicrobial agents (Shaikh and Fatima, 2015; Alam et al., 2022).

The World Health Organization (WHO) has identified over 20,000 species of medicinal plants as potential sources for new drug development (Srinivasan and Jayaprakasha, 2001), with more than 100 countries establishing regulations for their use. To date, over 1,340 plants have been recognized for their antimicrobial properties, and more than 30,000 antimicrobial compounds have been isolated from plants (Tajkarimi and Ibrahim, 2010; Vaou et al., 2021a). Desert-adapted plants are emerging as valuable sources of antimicrobial agents due to their remarkable ability to thrive in extreme environmental conditions, such as intense heat, high ultraviolet (UV) radiation, and scarce water. These harsh conditions have led to the evolution of diverse secondary metabolites in these plants, including alkaloids, flavonoids, tannins, and terpenoids, which are essential for their defense against pathogens. Research has shown that these bioactive compounds possess significant antimicrobial properties, making desert plants a promising avenue for discovering new treatments to address antibiotic-resistant bacteria (El-Hilaly et al., 2020; Mahizan and Ismail, 2019). For example, *Jasonia candicans (J. candicans)*, *Cistanche tubulosa (C. tubulosa)*, *Moltkiopsis ciliata (M. ciliata)*, and *Thymelea hirsute (T. hirsute)* have demonstrated significant antimicrobial activities, reflecting their evolutionary adaptations to extreme environments. *J. candicans*, a plant endemic to arid regions, exhibits potent antimicrobial properties, particularly against Gram-positive bacteria, due to its rich content of essential oils and flavonoids (Khalid et al., 2020). Similarly, *C. tubulosa*, known for its use in traditional medicine, shows broad-spectrum antimicrobial activity, attributed to its complex phenolic compounds and polysaccharides, which have been effective against various bacterial and fungal pathogens (Shao et al., 2019). *M. ciliata*, another desert plant, contains bioactive compounds that inhibit microbial growth, highlighting its potential as an antimicrobial agent (Al-Snafi, 2021). Additionally, *T. hirsuta* has been found to possess substantial antimicrobial activity, particularly due to its terpenoids and flavonoids, which exhibit strong inhibitory effects against both Gram-positive and Gram-negative bacteria (Joudeh and Linke, 2018). These findings underscore the potential of desert-adapted plants as sources of novel antimicrobial agents, offering valuable alternatives to conventional antibiotics.

While plant extracts have demonstrated potential as antimicrobial agents due to their diverse bioactive compounds, their use as standalone treatments for infections has notable limitations. The chemical composition of plant extracts can vary significantly depending on species, growing conditions, and extraction methods, leading to inconsistent efficacy and dosing difficulties (Wink, 2022). Additionally, many plant-derived compounds are less potent than conventional antibiotics, often necessitating higher doses that can be impractical and may pose toxicity risks (Nikaido, 2020). Furthermore, plant-derived compounds frequently suffer from limited bioavailability due to poor absorption, rapid metabolism, and low solubility, which further diminishes their therapeutic effectiveness (Anwar et al., 2023). Despite these limitations, plant extracts can still be valuable in managing antimicrobial resistance when used in combination with antibiotics. Some plant compounds act as resistance-modifying agents by enhancing antibiotic efficacy through mechanisms such as inhibiting bacterial efflux pumps or biofilm formation, which can lower the required antibiotic doses and reduce adverse effects (Rosato et al., 2020). Synergistic effects between plant extracts and antibiotics have been observed in combinatorial therapies, effectively combating multidrug-resistant bacteria and restoring the potency of existing antibiotics (Vaou et al., 2021b). Therefore, this study aims to evaluate the antimicrobial, anti-inflammatory, and antioxidative effects of Egyptian desert-adapted plants extracts, both alone and in combination with commonly used antimicrobial drugs, against multidrug-resistant Gram-negative bacterial strains isolated from sepsis patients. This research, utilizing both *in vitro* and *in vivo* studies, highlights the potential of natural plant-based therapies as promising combinative agents with existing antimicrobial drugs in combating sepsis associated with Gram-negative infections.

## Materials and methods

### Sample collection and ethical approval

The blood samples (n=400) used in this study were initially collected for diagnostic purposes to facilitate patient care, not for research. These samples were not specifically obtained for the study. However, informed consent was obtained from all the participants to carry out this study and to maintain patient confidentiality, all clinical and laboratory data were anonymized. This study adhered to ethical standards approved by the Port-Said University Ethical Approval Committee, under approval number REC.PHARM.PSU/2023/21.

### Bacterial strains

The bacterial strains employed in this study were isolated from positive blood cultures routinely collected from patients with bloodstream infections (BSIs) at the Department of Microbiology, Al-Demerdash Hospital, Egypt. Initially, the Gram-negative isolates were identified using standard microbiological techniques, which included Gram staining, biochemical tests, and cultural characteristics in addition to API (Analytical Profile Index) 20E (BioMérieux, Mary l’Etoile, France). Subsequent confirmation of these isolates was achieved using the VITEK-2 automated identification system (BioMérieux, 2021), which offers enhanced accuracy and efficiency in bacterial identification. The standard strain *E. coli* ATCC 25922 was used as a control strain. To preserve these clinical strains for future research, they were stored in glycerol stocks at -70°C, a method proven to maintain bacterial viability and genetic integrity over extended periods (Kumar et al., 2016; Harris et al., 2019a). This storage technique ensures the stability of the strains and facilitates their use in subsequent studies, contributing to reliable and reproducible research outcomes. Furthermore, Genetic detection of specific Gram-negative bacterial genes using traditional PCR was applied identifying pathogens including *E. coli*, *P. aeruginosa*, and *A. baumannii* without sequencing. This approach involves amplifying unique gene sequences associated with these bacteria to confirm their presence. For *E. coli*, the *uidA* gene, which encodes β-glucuronidase, is commonly targeted as it serves as a reliable marker for this pathogen (Sokurenko et al., 1998). In the case of *P. aeruginosa*, the *pvdA* gene, which is involved in pyoverdine biosynthesis, is utilized for identification due to its specificity to this bacterium (Schweizer, 2003). For *A. baumannii*, the *blaOXA-51* gene, a prevalent carbapenemase gene, is frequently employed to detect this pathogen (Perez et al., 2007). The PCR process involves isolating bacterial DNA from samples, amplifying the target genes with specific primers, and visualizing the PCR products through gel electrophoresis. The presence of bands of the expected size on the gel indicates the presence of the target bacteria. This method offers a rapid, cost-effective, and reliable means of pathogen detection, facilitating timely diagnosis and treatment (Miller et al., 2009; Ghosh et al., 2012).

### Antibiotic sensitivity test of clinical bacterial strains

Clinical bacterial isolates were assessed for susceptibility to commonly prescribed antimicrobial agents for sepsis using the Kirby-Bauer disc diffusion method. In this procedure, antimicrobial discs were applied to Mueller-Hinton agar plates inoculated with bacterial isolates. The antimicrobial agents tested included: β-lactam agents with β-lactamase inhibitors such as Ampicillin/Sulbactam (SAM 10/10 µg) and Piperacillin/Tazobactam (TZP 100/10 µg); cephalosporins including Cefepime (FEP 30 µg), Cefotaxime (CTX 30 µg), Ceftazidime (CAZ 30 µg), and Ceftriaxone (CRO 30 µg); a cephalosporin and β-lactamase inhibitor combination, Ceftazidime/Avibactam (CAZ/AVI 30/20 µg); fluoroquinolones like Ciprofloxacin (CIP 5 µg); carbapenems including Ertapenem (ERT 10 µg), Imipenem (IPM 10 µg), and Meropenem (MEM 10 µg); aminoglycosides such as Amikacin (AK 30 µg); and Aztreonam (ATM 30 µg); and Colistin (CT 10 µg). Susceptibility and resistance were interpreted according to Clinical and Laboratory Standards Institute (CLSI) guidelines (CLSI, 2023; EUCAST, 2024). To confirm the results, additional antimicrobial susceptibility testing was performed to determine the minimum inhibitory concentrations (MICs) using the VITEK-2 automated system. Isolates were classified as multidrug-resistant (MDR) if they exhibited resistance to at least three different classes of antibiotics (Magiorakos et al., 2012).

### Molecular detection of antimicrobial resistance genes

In this study, several resistance genes were analyzed to evaluate their role in antibiotic resistance. *bla_CTX-M_* (Extended-Spectrum β-Lactamase), responsible for hydrolyzing various β-lactam antibiotics, including penicillins and cephalosporins (Pitout and Laupland, 2008). *bla_OXA-48_* encodes a carbapenemase that confers resistance to carbapenems, crucial in treating multidrug-resistant infections (Poirel et al., 2012). *bla_NDM_* (New Delhi Metallo-β-Lactamase) imparts resistance to nearly all β-lactams, including carbapenems, complicating treatment options (Yong et al.., 2019a). *bla_SHV_* is associated with ESBL production, leading to resistance against penicillins and cephalosporins (Paterson and Bonomo, 2005). *bla_TEM_* is a widely known β-lactamase gene contributing to resistance primarily to penicillins and some cephalosporins (Datta and Hughes, 1983). These genes collectively highlight the critical challenge of antibiotic resistance and underscore the need for continued surveillance and novel treatment strategies.

For the PCR detection of resistance genes in bacterial isolates, total DNA was extracted from each isolate using the JET Genomic DNA Purification Kit (Thermo Scientific, USA) according to the manufacturer’s instructions. Following the extraction of DNA, the PCR amplification was conducted using primers and cycling conditions as specified in the protocols outlined in previous literature (**Table 1**). This approach adhered to established methods to ensure accuracy and reproducibility in the detection of specific genes. The primer sequences and cycling parameters were selected based on validated protocols from prior studies, which have been demonstrated to effectively amplify the target genes of interest (**Table 1**). To ensure the specificity of these primers, they were analyzed using NCBI Primer-BLAST, a tool available at the NCBI website (https://www.ncbi.nlm.nih.gov/guide/data-software/). This process ensures that the primers effectively target the intended genes and minimize non-specific amplification.

**Table 1:** Nucleotide sequences and amplicon size of oligonucleotides primers used for PCR detection of resistance genes of the tested Gram-negative bacterial isolates.

Gene	Sequences	Annealing Temperature	Amplicon Size (bp)	References
*bla*_CTX-M_	F: ATGGTTAAAAAATCACTGCGYC R: TTACAAACCGTCGGTG	51°C	876	Patel, 2015
*bla*_OXA-48_	F: GCGTGGTTAAGGATGAACAC R: CATCAAGTTCAACCCAACCG	45°C	438	Poirel and Nordmann, 2006
*bla*_NDM_	F: GGTTTGGCGATCTGGTTTTC R: CGGAATGGCTCATCACGATC	45°C	621	Yong et al., 2019b
*bla*_SHV_	F-AGGATTGACTGCCTTTTTG R-ATTTGCTGATTTCGCTCG	94°C	392	Paterson and Bonomo, 2005
*bla*_TEM_	F-TTGGGTGCACGAGTGGGTTA R-TAATTGTTGCCGGGAAGCTA	95°C	465	Kim et al., 2015

### Collection and extraction of the desert-adapted plants materials

Four species of desert-adapted plants, traditionally utilized for treating severe bacterial infections, were collected from their natural habitat in the Egyptian desert. These plants, detailed in **Table 2**, were identified by the Herbarium of the Desert Research Center in Egypt. For each plant, 300 grams of shade-dried, coarsely powdered aerial parts were extracted using 70% ethanol through a maceration process. The evaporation of ethanol was confirmed by concentrating the resulting extracts to dryness under reduced pressure using a rotary evaporator set at 40°C. This process effectively removed the ethanol from the extracts, leaving behind the concentrated plant material, which was then stored at 4°C until further use (Ghaly et al., 2023).

**Table 2:** Selected plant extracts, their families, and traditional use.

Plant name	Family	Traditional use	References
*Jasonia candicans* Del. Borsh	*Asteraceae*	Antimicrobial activity Anti-inflammatory	Francisco Les et al., 2021; Hammerschmidt et al., 1993
*Cistanche tubulosa* (Schrenk) Hook.f.	*Orobanchaceae*	Antibacterial activity Enhances immunity. Antioxidant Neuroprotection Hepatoprotection	Li et al., 2016 Yuan et al., 2018
*Moltkiopsis ciliata* (Forssk.) I. M. Johnst	*Boraginaceae*	Antimicrobial Antioxidant Anti-inflammatory Anticancer	Abdel-Hak et al., 2022
*Thymelea hirsuta*(L.) Endl.	*Thymelaeaceae*	Anti-inflammatory Neuroprotective Antioxidant	Marmouzi et al., 2021

### Preliminary screening of plant extracts

The antibacterial efficacy of the plant extracts was evaluated using the agar well diffusion method. Bacterial cultures, standardized to a 0.5 McFarland turbidity equivalent, were evenly spread on sterile Mueller-Hinton agar plates to achieve uniform bacterial distribution. Plant extracts, dissolved in dimethyl sulfoxide (DMSO) (10 mg/mL), were then introduced into wells drilled into the agar at a volume of 100 µL per well. The plates were incubated at 37°C for 24 hours to allow for bacterial growth and interaction with the extracts. After incubation, the diameters of the inhibition zones surrounding each well were measured in millimeters to assess the antimicrobial activity of the extracts (Murray et al., 2016; Clinical and Laboratory Standards Institute, 2023). A positive control well containing colistin and a negative control well containing DMSO were included in the assay for comparison.

### Determination of minimum inhibitory concentration of plant extracts

The minimum inhibitory concentrations (MICs) of plant extracts against identified Gram-negative strains were determined using the broth microdilution method. Plant extracts were initially dissolved in 10% DMSO to achieve a final concentration of 1 mg/mL in the culture broth. Serial 1:2 dilutions were then performed to obtain a range of concentrations from 1 mg/mL to 0.0156 mg/mL. Each dilution (100 µL) was added to a 96-well plate, along with negative and positive controls: culture broth with DMSO and culture broth with colistin, respectively. Each well, including the test and growth control wells, was inoculated with 5 µL of a bacterial suspension at a concentration of 10^5^ CFU/mL ( (Murray et al., 2016; Eloff, 1998).

All experiments were performed in triplicate and the microdilution trays were incubated at 37°C for 18 h. After incubation, the minimum inhibitory concentration (MIC) was determined by measuring the optical density of each well using a spectrophotometer, similar to the principles used in an ELISA assay to assess bacterial growth inhibition. MIC values were defined as the lowest concentration of each plant extract, which completely inhibited microbial growth (Valgas et al., 2007).

### Evaluating the efficacy of the most effective desert-adapted plant extracts in combination with amikacin, a commonly prescribed antimicrobial agent

The checkerboard method is an established approach for determining the fractional inhibitory concentration (FIC) index, which assesses the interaction between a plant extract and a common antimicrobial drug (amikacin). This technique involves creating serial dilutions of both the plant extract and the antimicrobial drug, which are then combined in a 96-well microtiter plate, with each well containing a different concentration of the extract and drug. After adding a standardized bacterial suspension to each well, the amikacin-resistant Gram-negative bacteria, which exhibited multidrug-resistant (MDR) patterns (40 isolates with higher multiply antibiotic indices), were incubated at 37°C for 18-24 hours to facilitate bacterial growth and interaction with the compounds. The effectiveness of the plant extract and drug, both individually and in combination, is evaluated by measuring the minimum inhibitory concentration (MIC) for each in a triplicate manner. The FIC index is calculated using the formula: FIC index = (MIC of plant extract in combination/MIC of plant extract alone) + (MIC of antimicrobial drug in combination/MIC of antimicrobial drug alone). This index indicates the nature of the interaction—whether synergistic (FIC index ≤ 0.5), additive (FIC index > 0.5 but ≤ 1), or antagonistic (FIC index > 1). By revealing potential synergistic or antagonistic effects, the checkerboard method aids in optimizing combination therapies and understanding how plant extracts can enhance antimicrobial treatment (Odds, 2003; Tamma et al., 2012).

### Determination of the anti-inflammatory activity of tested plant extracts

The inhibition of Cyclooxygenase-1 (COX-1) and COX-2 enzymes by the tested plant extracts was assessed using an ELISA-based method in triplicate manner. Plant extracts were dissolved in 10 µL of DMSO to create a series of stock solutions with concentrations ranging from 50 mmol/L to 500 pmol/L. Celecoxib served as the positive control. The inhibition of recombinant COX-1 and COX-2 enzymes was measured using a Cayman Human (EIA) kit (Catalog No. 560131, Cayman Chemicals Inc, MI, USA) and analyzed with a Robomik P2000 ELISA reader at 450 nm. The effectiveness of the plant extracts was quantified by determining the mean concentration required to achieve 50% enzyme inhibition (IC_50_) ± standard deviation. Additionally, the COX-2 selectivity index (SI) was calculated as the ratio of IC_50_(COX-1) to IC_50_(COX-2) and compared to that of celecoxib to evaluate selectivity (Baek et al., 2021).

### Determination of antioxidant activity of tested plant extracts using DPPH assay

The antioxidant activity (AA%) of each substance was evaluated using the 2,2-diphenyl-1-picryl-hydrazyl-hydrate (DPPH) free radical assay, as described by Boly et al. (2016). In this assay, 100 μL of freshly prepared DPPH reagent (0.1% in ethanol) was mixed with 100 μL of each sample, which had been dissolved in ethanol, in a 96-well plate (n=6). The reaction mixture was incubated at room temperature for 30 minutes in the dark. The reduction in radical activity was monitored by measuring the absorbance at 540 nm using a microplate reader (FluoStar^®^ Omega, WWU, Münster, Germany), with ethanol serving as the blank. The percentage of scavenging activity was calculated using the formula:


$$\text{AA}\%=100-[\left({\text{Abs}}_{\text{sample}}-{\text{Abs}}_{\text{blank}}\right)\times 100/{\text{Abs}}_{\text{control}}$$

Where Abs_control_ is the absorbance at 540 nm of 100μM DPPH solution without addition of the extract, Abs_sample_ is the absorbance at 540nm of 100μM DPPH with 5–100 μg/mL of sample. Trolox was used as a reference standard. The IC_50_ value, which represents the concentration of extract required to reduce DPPH by 50%, was determined by plotting the percentage inhibition against concentration using GraphPad Prism 5^®^ (Chen et al., 2013).

### *In Vivo* evaluation of synergistic interactions between the highest effective plant extract and the most common prescribed antimicrobial agent in a murine model

#### Experimental design and animal model

An experimental septicemic mouse model, as depicted in **Supplementary Figure S1**, was developed to evaluate the promising antimicrobial effects of *J. candicans* extract on sepsis induced by 4 multi-drug resistant (MDR) isolates with the highest multi-antibiotic resistance (MAR) index including *E. coli*, *K. pneumoniae*, *A. baumannii*, and *P. aeruginosa*, following ethical guidelines from the Port-Said University Ethical Committee. After a one-week quarantine, 130 male BALB/c mice (20–25 g) were randomly assigned to thirteen groups (n = 10 per group): Group A served as the general negative control (unchallenged and untreated); Group B was the positive control for *K. pneumoniae* (challenged and untreated); Group C included *K. pneumoniae*-challenged mice treated with amikacin; Group D received a combination of *J. candicans* extract and amikacin after *K. pneumoniae* challenge; Group E was the positive control for *E. coli* (challenged and untreated); Group F consisted of *E. coli*-challenged mice treated with amikacin; Group G received *J. candicans* extract and amikacin after *E. coli* challenge; Group H served as the positive control for *A. baumannii* (challenged and untreated); Group I included *A. baumannii*-challenged mice treated with amikacin; Group J received a combination of *J. candicans* extract and amikacin after *A. baumannii* challenge; Group K was the positive control for *P. aeruginosa* (challenged and untreated); Group L consisted of *P. aeruginosa* challenged mice treated with amikacin; and Group M received *J. candicans* extract and amikacin after *P. aeruginosa* challenge. All mice were housed in a specific pathogen-free (SPF) environment with controlled conditions: temperature 20–22°C, humidity 40–70%, and a 12-hour light/dark cycle, and were anesthetized using isoflurane inhalation.

#### Establishment of acute sepsis models

To establish the sepsis model, all groups except the negative control group were injected intraperitoneally with bacterial suspensions. Groups B-D received 0.04 mL of *K. pneumoniae* containing 10^9^ CFU/mL; Groups E-G were administered 2 to 5 × 10^7^ CFU of *E. coli*; Groups H-J were given 0.5 mL of *A. baumannii* suspension containing 10^7^ CFU/mL; and Groups K-M received 2 × 10^10^ CFU of *P. aeruginosa* in 100 μL of saline. The negative control group received an equivalent volume of saline (pH 7.4). Antimicrobial treatments began 2 hours post-infection, with amikacin administered intraperitoneally at 15 mg/kg every 12 hours for three days. The dosing regimen was determined based on pharmacokinetic and pharmacodynamic data from previous studies (Song et al., 2009; Bretonnière et al., 2010). In groups treated with the combination of *J. candicans* extract and amikacin, the treatment was also administered intraperitoneally every 12 hours for three days at sub-minimum inhibitory concentration (sub-MIC) levels (0.5 MIC) (Verweij et al., 2004; Singh et al., 2022; Liu et al., 2022; Harris et al., 2019b; Cirioni et al., 2007). Finally, we conducted regular monitoring of the animals, assessing key health indicators, including body weight, activity levels, and clinical signs of infection (such as lethargy, ruffled fur, and reduced food intake) and we incorporated this results in our new manuscript.

#### Histopathological analysis

Four days after treatment, blood samples were collected from the ophthalmic vein plexus of the mice to evaluate biological indices. Following this, six mice from each group were randomly selected for histopathological examination of the lung and spleen. For the histopathological analysis, tissues from the spleen, and lungs were fixed in formalin, embedded in paraffin, sectioned at 5 µm thickness, and stained with hematoxylin and eosin (H&E) for examination. Histological specimens were then analyzed under a light microscope, and lesions were assessed qualitatively and quantitatively. Of note, 10 microscopic fields per sample were examined for both splenic and lung tissues across each challenged and unchallenged mice group. Fields were selected randomly to ensure unbiased analysis and provide a comprehensive assessment of tissue alterations in response to challenges. Lesions in the spleen and lungs were scored according to their severity: (++++): maximum severity, (+++): marked severity, (++): moderate severity, (+): mild severity, and (0): no pathological changes. The mice were euthanized by cervical dislocation for these assessments.

## Results

### Prevalence and antimicrobial resistance patterns of gram-negative bacteremia

A total of 400 blood samples were collected for blood culture analysis, resulting in the identification of 233 clinical bacterial isolates as Gram-negative pathogens, yielding a prevalence rate of 58.25%. Among these Gram-negative isolates, *E. coli* and *K. pneumoniae* were the predominant pathogens, with 113 isolates (48.5%) and 90 isolates (38.6%), respectively. The next most common pathogens were *A. baumannii*, represented by 16 isolates (6.9%), and *P. aeruginosa*, with 14 isolates (6%).

**Figure 1:**
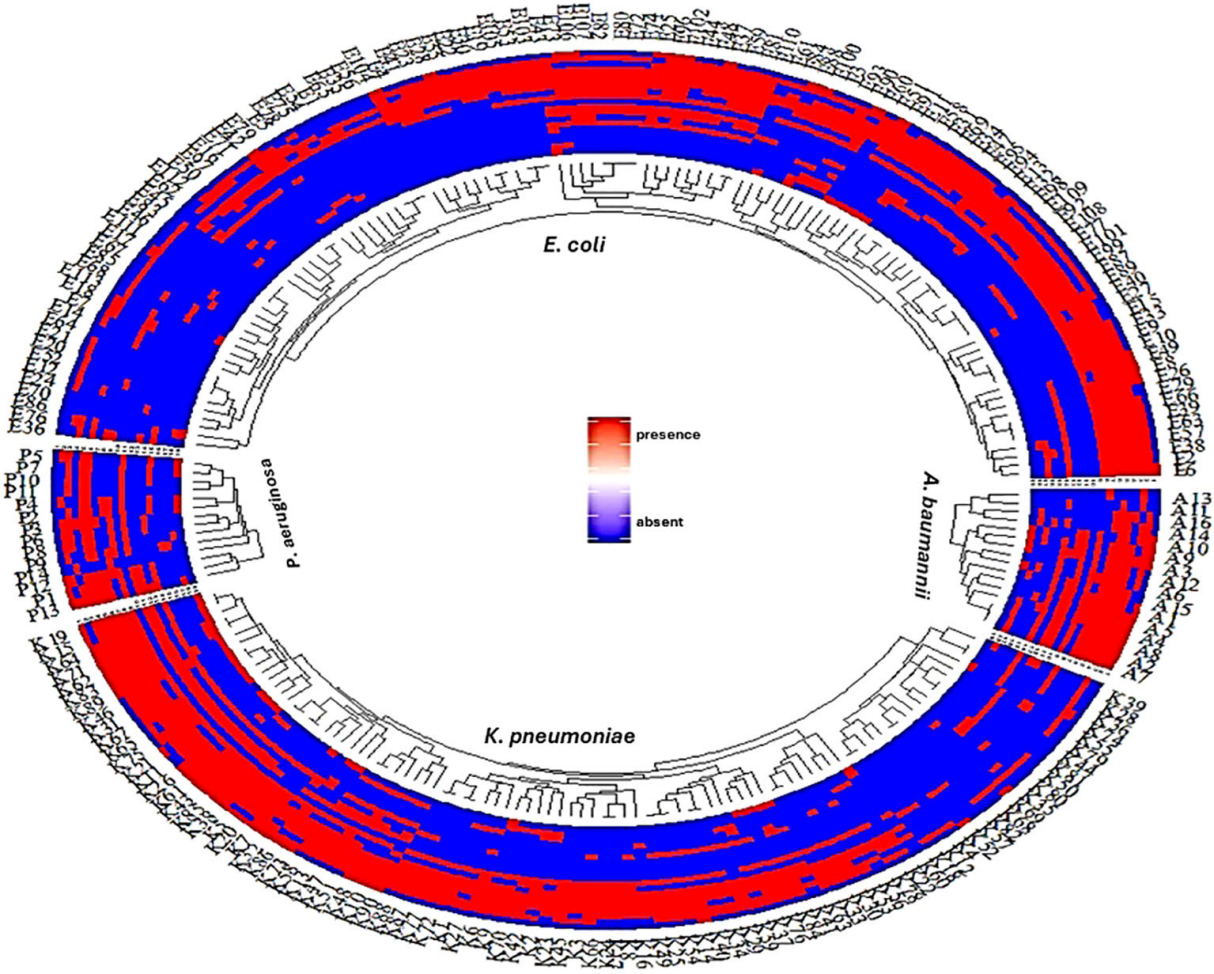
A fan dendrogram illustrating the distribution of Gram-negative bacteria based on their antimicrobial resistance profiles and associated resistance genes. This figure showed the distribution of antimicrobial resistance and resistance gene profiles among the investigated Gram-negative bacterial species: *E. coli*, *K. pneumoniae*, *P. aeruginosa*, and *A. baumannii*. The inner circle shows clustering of strains based on species, while the outer rings indicate the presence (red) or absence (blue) of resistance genes. The consistent red regions suggest widespread presence of resistance genes across species, particularly for *E. coli* and *K. pneumoniae*. In contrast, *P. aeruginosa* and *A. baumannii* show a more heterogeneous pattern of resistance gene distribution. Line1: AK, line2: ATM, line3: SAM, line4: FEB, line5: CTX, line6: CRO, line7: CAZ, line8: CAZ/AVI, line9: CIP, Line10: CT, line11: TZP, line12: IPM, line: ETP, line14: MEM, line15: *bla_OXA_*, line16: *bla_NDM_*, line17: *bla_SHV_*, line18: *bla_CTX_*, line19: *bla_TEM_*,.

Analysis of the antimicrobial resistance patterns among these clinical isolates revealed a significant burden of multidrug resistance (MDR), as 185 isolates (79.4%) demonstrated resistance to 3 or more antimicrobial classes. Notably, *P. aeruginosa* had the highest MDR prevalence (92.8%). This was followed by *A. baumannii*, with 81.2% of isolates showing MDR patterns. Additionally, a high prevalence of MDR was observed among *E. coli* and *K. pneumoniae*, with rates of 77.9% and 78.9%, respectively. The distribution of antimicrobial resistance and resistance gene profiles among the investigated Gram-negative bacterial species: *E. coli*, *K. pneumoniae*, *P. aeruginosa*, and *A. baumannii*. were observed in **Figure 1**. Our results indicated a varied distribution and heterogenous patterns of antimicrobial resistance and resistance gene profiles among different Gram-negative bacterial species. The results further revealed a heterogeneous pattern of antimicrobial resistance gene distribution, particularly in *Pseudomonas aeruginosa* and *Acinetobacter baumannii*. In contrast to *E. coli* and *K. pneumoniae*, where antimicrobial resistance and resistance genes were more consistently distributed among strains, *P. aeruginosa* and *A. baumannii* exhibit significant variability in the presence and absence of resistance genes, reflecting low clonality and high heterogeneity within these isolates.

**Figure 2:**
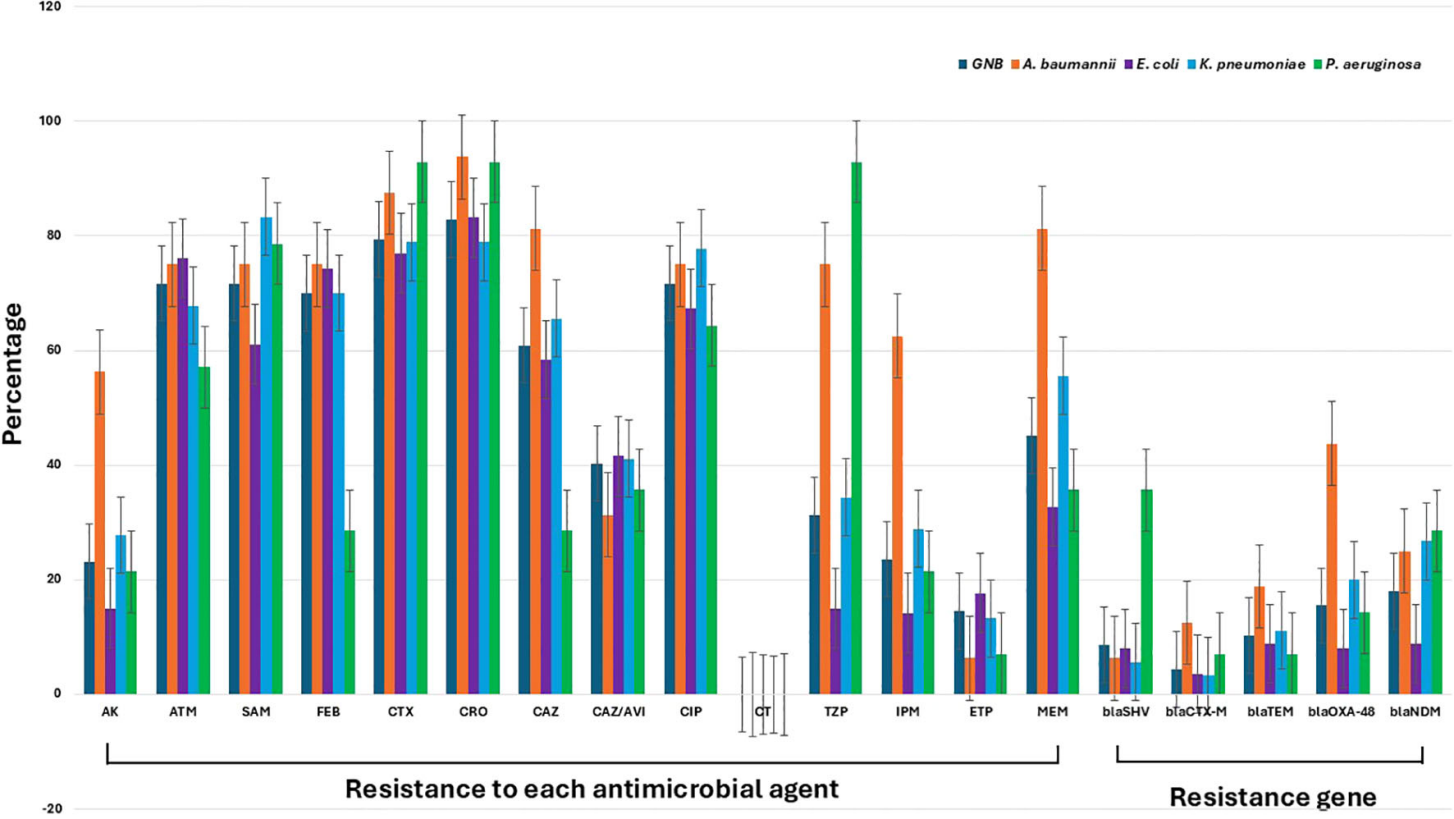
Antimicrobial resistance patterns and resistance gene prevalence among Gram-negative bacteria isolated from septicemic patients. The figure shows the percentage resistance of various Gram-negative pathogens, including *A. baumannii*, *E. coli*, *K. pneumoniae*, and *P. aeruginosa*, to a range of antimicrobial agents: amikacin (AK), aztreonam (ATM), ampicillin-sulbactam (SAM), cefepime (FEB), cefotaxime (CTX), ceftriaxone (CRO), ceftazidime (CAZ), ceftazidime-avibactam (CAZ/AVI), ciprofloxacin (CIP), colistin (CT), piperacillin-tazobactam (TZP), imipenem (IPM), ertapenem (ETP), and meropenem (MEM). The data also include the prevalence of key resistance genes: *bla_SHV_, bla_CTX-M_, bla_TEM_, bla_OXA-48_*, and *bla_NDM_*. The gray columns represent the overall resistance profile of Gram-negative bacteria.

The results for antimicrobial resistance across all Gram-negative bacteria, represented in **Figure 2**, revealed substantial resistance to a wide range of antimicrobial agents. Conversely, all tested isolates demonstrated complete susceptibility to colistin, an antibiotic considered as a last-resort treatment for MDR infections. Overall, all investigated isolates displayed notably high resistance rates across various antibiotics. For amikacin, the isolates showed a moderate resistance rate of approximately 30%. However, resistance to aztreonam and ampicillin-sulbactam was significantly higher, at around 70-80%, reflecting a broad decline in the efficacy of these agents. Resistance to cefepime, cefotaxime, ceftriaxone, and ceftazidime was consistently elevated, with rates in the 70-80% range, underscoring the substantial challenges in treating septicemic patients with these commonly used beta-lactam antibiotics.

Of note, the resistance patterns among the detected Gram-negative species indicate that, for several antimicrobial, including aztreonam, ampicillin-sulbactam, cefotaxime, ceftriaxone, ceftazidime/avibactam, ciprofloxacin, ertapenem, and temocillin, the levels of resistance did not reach statistical significance, with *P-values* exceeding 0.05. This suggests that resistance to these antibiotics may not be markedly different across the groups analyzed. In contrast, the resistance to other antimicrobial agents showed statistically significant differences (*P< 0.05*), indicating that these other drugs are likely experiencing varied effectiveness due to differing resistance levels among the bacterial species tested. These findings highlight specific antibiotics that may be more or less effective in treating infections caused by these Gram-negative organisms, underscoring the importance of ongoing surveillance to guide antibiotic selection and optimize treatment strategies.

When focusing on specific pathogens, *A. baumannii*, *E. coli*, *K. pneumoniae*, and *P. aeruginosa*, the data demonstrated high levels of resistance across most tested antimicrobial agents, with *A. baumannii* generally showing the highest resistance rates. For amikacin, *A. baumannii* exhibited resistance near 60%, whereas *E. coli*, *K. pneumoniae*, and *P. aeruginosa* showed lower resistance levels, typically around 20-40%. Resistance to aztreonam and ampicillin-sulbactam was uniformly high across all pathogens, ranging from 70-90%, with *A. baumannii* and *K. pneumoniae* particularly high resistance levels (**Figure 2**). The presence of resistance genes such as *blaSHV, blaCTX-M, blaTEM, blaOXA-48*, and *blaNDM* highlighted the molecular mechanisms driving resistance in these pathogens. Notably, *blaNDM* was highly prevalent in *A. baumannii* and *K. pneumoniae*, contributing significantly to the high levels of carbapenem resistance observed as shown in **Figure 2**. Except for the presence of the *blaTEM* gene, all other resistance genes showed significant variation among the Gram-negative species detected (*P< 0.05*). These findings underscored the severe antimicrobial resistance challenges posed by these Gram-negative pathogens in septicemic patients, emphasizing the urgent need for alternative therapeutic strategies and stringent antimicrobial stewardship practices.

### Antimicrobial activity of medicinal plants and their activity against tested bacteria

Ethanol extracts from medicinal plants were assessed for antimicrobial activity against MDR Gram-negative bacteria from septic patients. The agar diffusion assay revealed significant antibacterial effects, with inhibition zones ranging from 13 to 25 mm. *J. candicans* emerged as the most effective, showing inhibition zones of 18–25 mm, particularly strong against *A. baumannii* (25 mm) and *E. coli* (19 mm), and effective against *P. aeruginosa* and *K. pneumoniae* (18 mm each). *M. ciliata* also showed notable activity, especially against *A. baumannii* (20 mm) and *E. coli* (16 mm). In contrast, *C. tubulosa* and *T. hirsuta* exhibited more variable results; *T. hirsuta* had significant inhibition against *E. coli* (15 mm) and *A. baumannii* (14 mm), while *C. tubulosa* was least effective, particularly against *E. coli* (13 mm).

Broth microdilution testing revealed that *J. candicans* exhibited the highest antimicrobial potency among the tested plant extracts (**Supplementary Table S1**). It demonstrated the lowest MIC values, with 250 µg/mL against most *E. coli* isolates, and 500 µg/mL against most *K. pneumoniae, A. baumannii*, and *P. aeruginosa* isolates. In contrast, *C. tubulosa* showed comparatively higher MIC values, reflecting a reduced effectiveness: 500 µg/mL against most *E. coli* and *K. pneumoniae* isolates, 1000 µg/mL against most *P. aeruginosa*, and *A. baumannii* isolates. *M. ciliata* exhibited variable efficacy, with MIC values of 500 µg/mL against most *E. coli* and 1000 µg/mL for most *A. baumannii*, *K. pneumoniae*, and *P. aeruginosa* isolates. *T. hirsuta* had the highest MIC values across all tested bacteria, with 1000 µg/mL against most investigated isolates. Overall, *J. candicans* demonstrated the strongest antimicrobial potential, followed by *M. ciliata*, indicating its superior effectiveness in inhibiting the growth of these Gram-negative bacteria.

### Combined action of common prescribed antibiotics and the most effective plant extract

The checkerboard assay, as shown in **Supplementary Table S1**, assessed the combined effect of the widely prescribed antibiotic amikacin and *J. candicans* extract, demonstrating a synergistic interaction against all investigated amikacin-resistant, multidrug-resistant (MDR) Gram-negative bacteria (40 isolates). The combination of *J. candicans* with amikacin resulted in a notable reduction in the MIC values for both agents, with a FICI ≤ 0.5, indicating a significant enhancement in antimicrobial efficacy. Specifically, this combination therapy substantially lowered the MIC values for *E. coli* and, to a lesser extent, for *K. pneumoniae*, *A. baumannii*, and *P. aeruginosa*. These results suggest that *J. candicans* and amikacin work synergistically to inhibit bacterial growth more effectively than either agent alone. Moreover, this synergistic interaction highlights the potential of integrating *J. candicans* extract with amikacin as a promising therapeutic strategy to improve treatment outcomes for infections caused by resistant Gram-negative bacteria. Interestingly, over 50% of the amikacin-resistant strains were converted to sensitive phenotypes by this combination, further emphasizing the therapeutic potential of this approach.

### 2 Anti-inflammatory activity of plant extracts using invitro COX-1/COX-inhibition assay

Our results indicated that *J. candicans* extract exhibited the most potent inhibitory activity against both COX-1 and COX-2 enzymes, with IC_50_ values significantly lower than those of the standard drug, Celecoxib (IC_50_: 0.28 μg/mL for COX-1 and 1.11 μg/mL for COX-2). Specifically, *J. candicans* demonstrated exceptional efficacy in inhibiting these enzymes, suggesting its potential as a therapeutic agent for inflammatory conditions. In addition, the extracts of *C. tubulosa* and *T. hirsuta* also exhibited noteworthy inhibitory effects on both COX-1 and COX-2, with IC_50_ values that closely approached those of Celecoxib, as detailed in **Table 3**. Importantly, all four plant extracts showcased a COX-2 selectivity index comparable to that of Celecoxib, ranging from 0.23 to 0.28. This selectivity indicates a preference for inhibiting COX-2 over COX-1, which is advantageous for minimizing gastrointestinal side effects often associated with non-selective COX inhibitors. The ability of these extracts to selectively target COX-2 further emphasizes their potential as valuable anti-inflammatory agents. Overall, the findings suggest that these plant extracts, particularly *J. candicans*, not only provide strong anti-inflammatory effects but also present a promising alternative to conventional NSAIDs. Further investigation into their mechanisms of action and clinical applications is warranted to fully explore their therapeutic potential in managing inflammation and related conditions.

**Table 3:** *In vitro* COX-1 IC_50_, COX-2 IC_50_, and COX-2 Selectivity Index (SI) data for tested plant extracts in comparison to Celecoxib (IC_50_ in μg/ml).

Plant Extracts	COX1- IC_50_ (µg/ml) [mean IC_50_ values ± SD]	COX2- IC_50_ (µg/ml) [mean IC_50_ values ± SD]	COX-2 Selectivity index (SI)
*Jasonia candicans*	0.28 ± 0.0006	1.11 ± 0.1	0.23
*Cistanche tubulosa*	0.31 ± 0.0012	1.32 ± 0.15	0.23
*Moltkiopsis ciliata*	0.44 ± 0.0015	1.57 ± 0.4	0.28
*Thymelia hirsuta*	0.3 ± 0.00006	1.19 ± 0.1	0.25
Celecoxib	0.34	1.2	0.28

### Antioxidant activity of tested plant extracts

The DPPH assay was utilized to assess the scavenging activity of the tested plant extracts alongside the standard antioxidant, Torolox. This assay determines the concentration of the extract needed to reduce 50% of the initial DPPH radical. Among the plant extracts, *J. candicans* demonstrated the highest DPPH free radical scavenging activity, with a notably low IC_50_ value of 71.97 μg/mL, surpassing *C. tubulosa*, which had an IC_50_ value of 97.44 μg/mL. In contrast, *M. ciliata* and *T. hirsute* exhibited the lowest scavenging activity, with IC_50_ values of 156 μg/mL and 193 μg/mL, respectively. For comparison, the reference antioxidant Torolox showed an IC_50_ value of 56.82 μg/mL. These findings highlight that while *J. candicans* and *C. tubulosa* display notable antioxidant activity, Torolox remains the most effective agent for scavenging DPPH radicals, outperforming the plant extracts.

### Assessment of clinical signs as indicators of disease severity in a septicemia model

Data collected over the study period showed that animals in the control group exhibited significant weight loss and clinical signs of illness, which are indicative of severe septicemia. In contrast, animals receiving treatment with the combination of desert plant extracts and amikacin demonstrated less pronounced weight loss and a notable improvement in clinical signs, suggesting a better overall health status. Specifically, animals treated with the combination showed an average weight loss of approximately 5% compared to 15% in the amikacin-only group, indicating enhanced recovery with the extract. Additionally, the survival rate in the combination treatment group was higher, with 80% of animals surviving until the end of the study, compared to 50% in the amikacin group alone.

### Histopathology results

**Figure 3:**
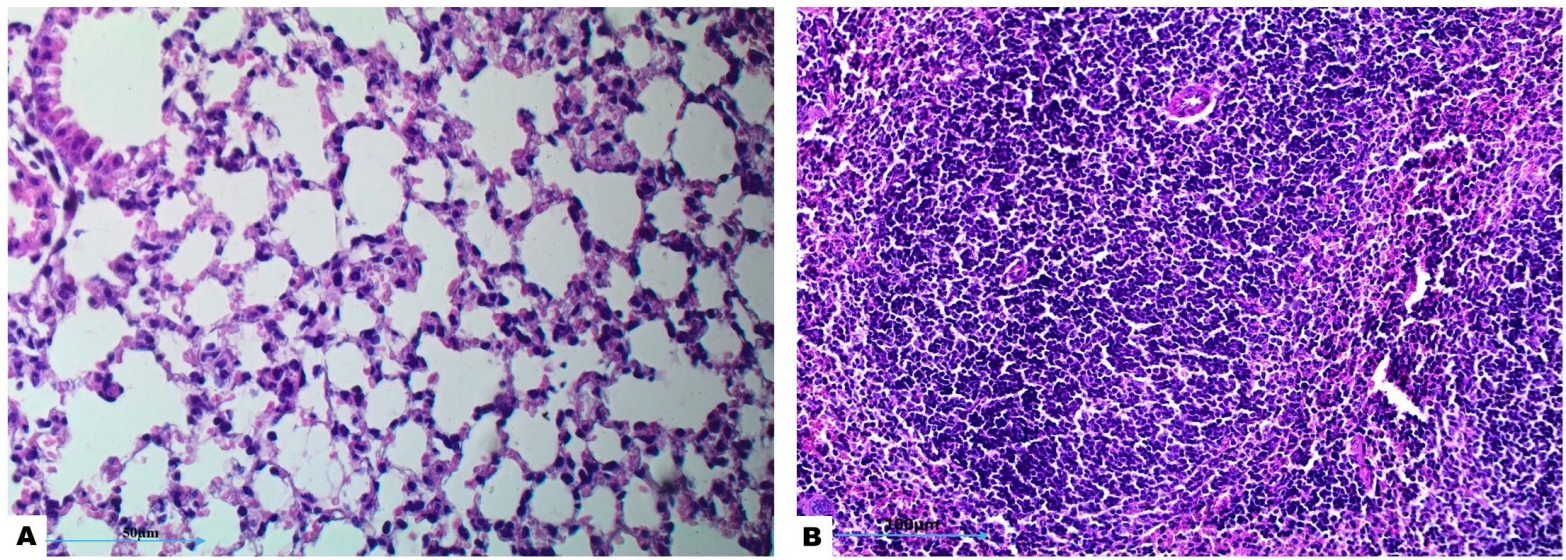
Histological examination of lung and splenic tissues from control negative (unchallenged and untreated) group exhibits normal histological features. **(A)** Normal alveolar structure observed under H&E staining at 400x magnification, and **(B)** normal splenic tissue observed under H&E staining at 200x magnification.

**Figure 4:**
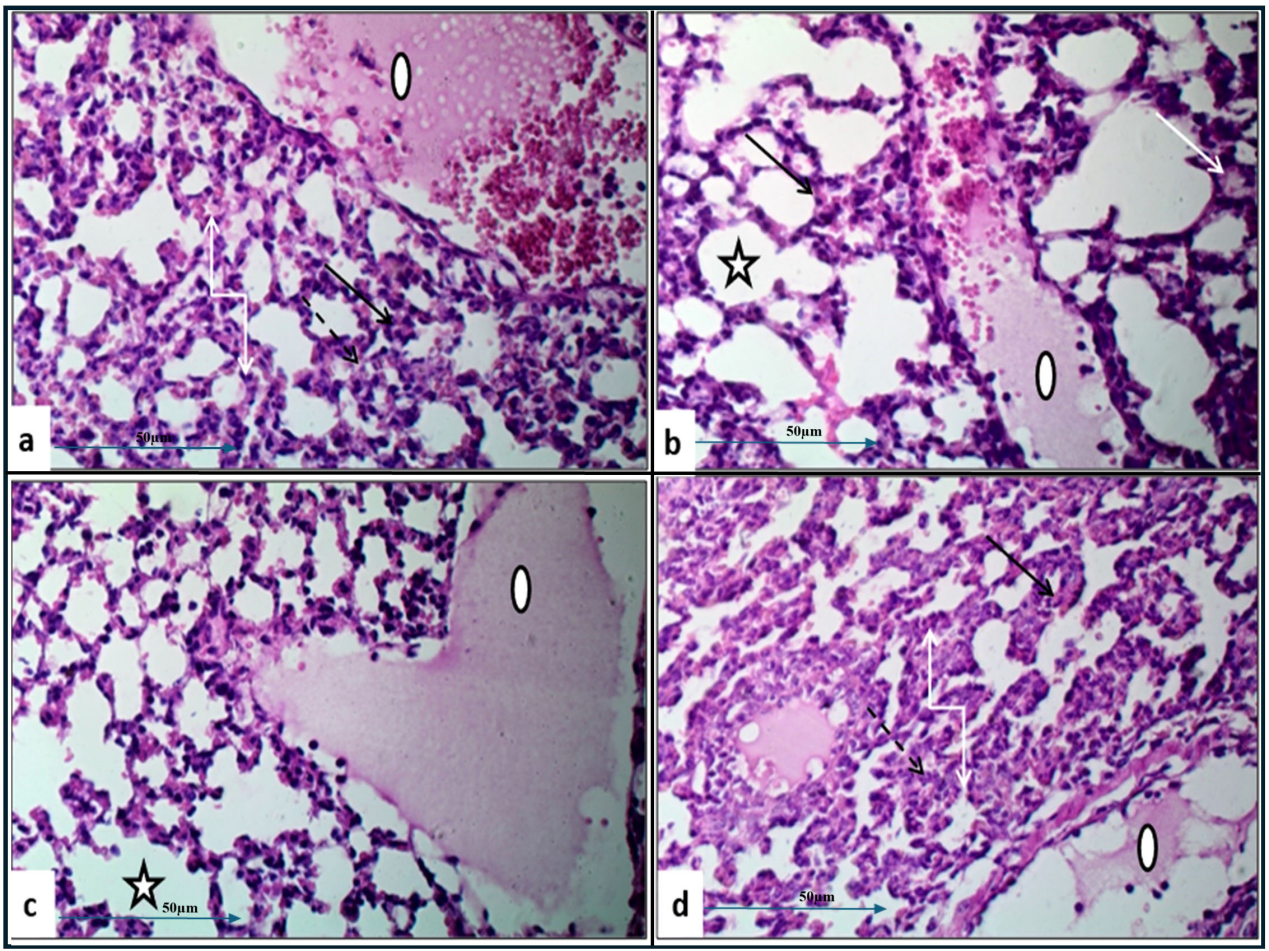
Histological examination of lung tissue from challenged untreated groups (H&E, 400x magnification). Histopathological analysis of untreated groups challenged with *A. baumannii* **(a)**, *E. coli* **(b)**, *K. pneumoniae* **(c)**, and *P. aeruginosa* **(d)** reveals alveolar wall collapse (truncated arrows), emphysema (asterisks), congestion (black arrows), hemorrhage/edema (white arrows), and pulmonary vasculature changes, including neutrophil infiltration (dashed arrows) and thrombus formation (circles).

**Figure 5:**
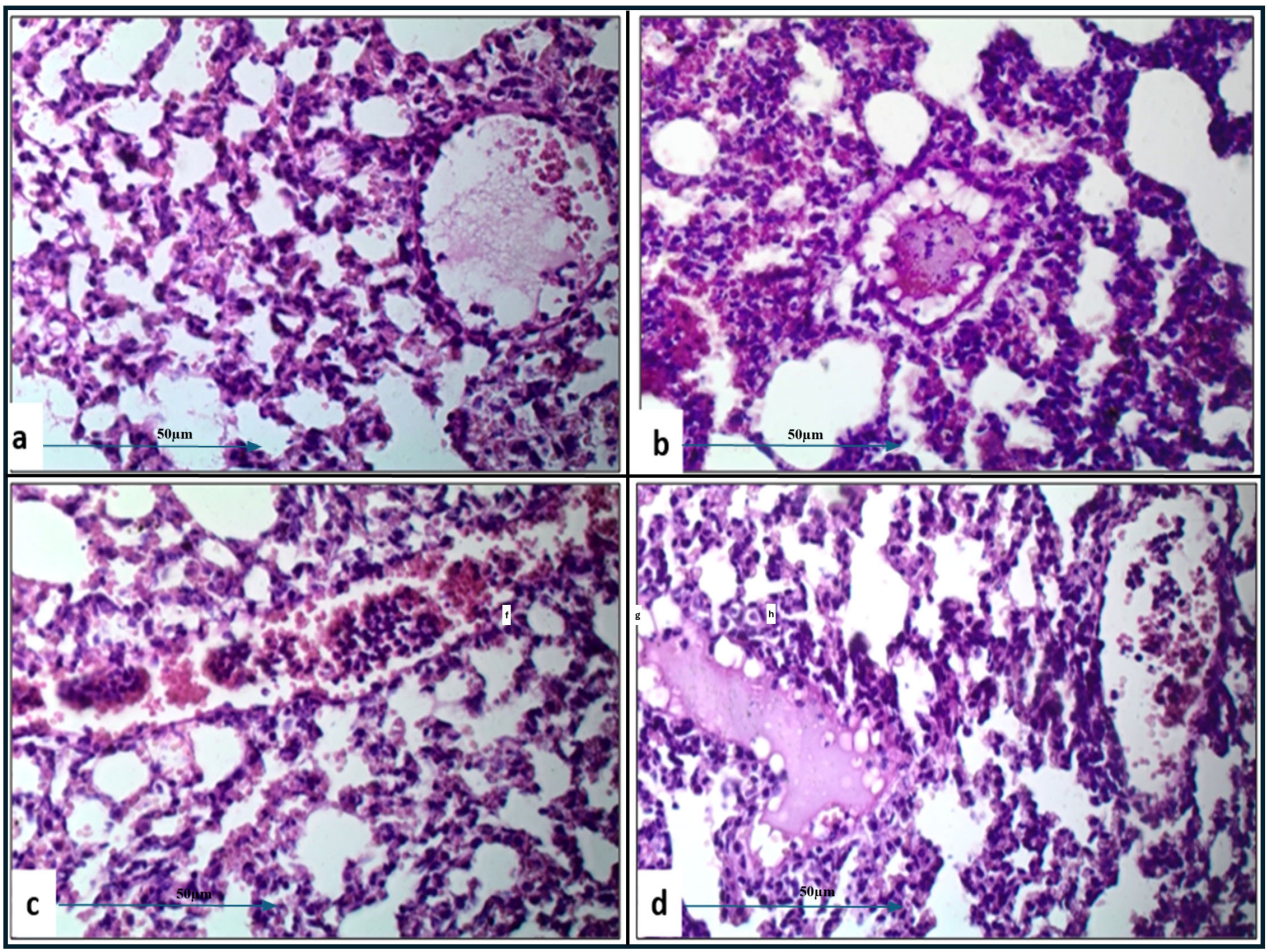
Histological examination of lung tissue from amikacin-treated challenged groups (H&E, 400x magnification) revealed alveolar wall collapse and congestion across all groups. Histopathological analysis of the amikacin-treated groups challenged with *A. baumannii* **(a)**, *E. coli* **(b)**, *K. pneumoniae* **(c)**, and *P. aeruginosa* **(d)** showed alveolar emphysema, which was pronounced in **(b, c)**, but moderate in **(a, d)**. Additionally, alveolar edema and hemorrhage were still observed in **(a–c)**. Changes in pulmonary vasculature, such as thrombus formation and neutrophil infiltration, were detected in all groups.

**Figure 6:**
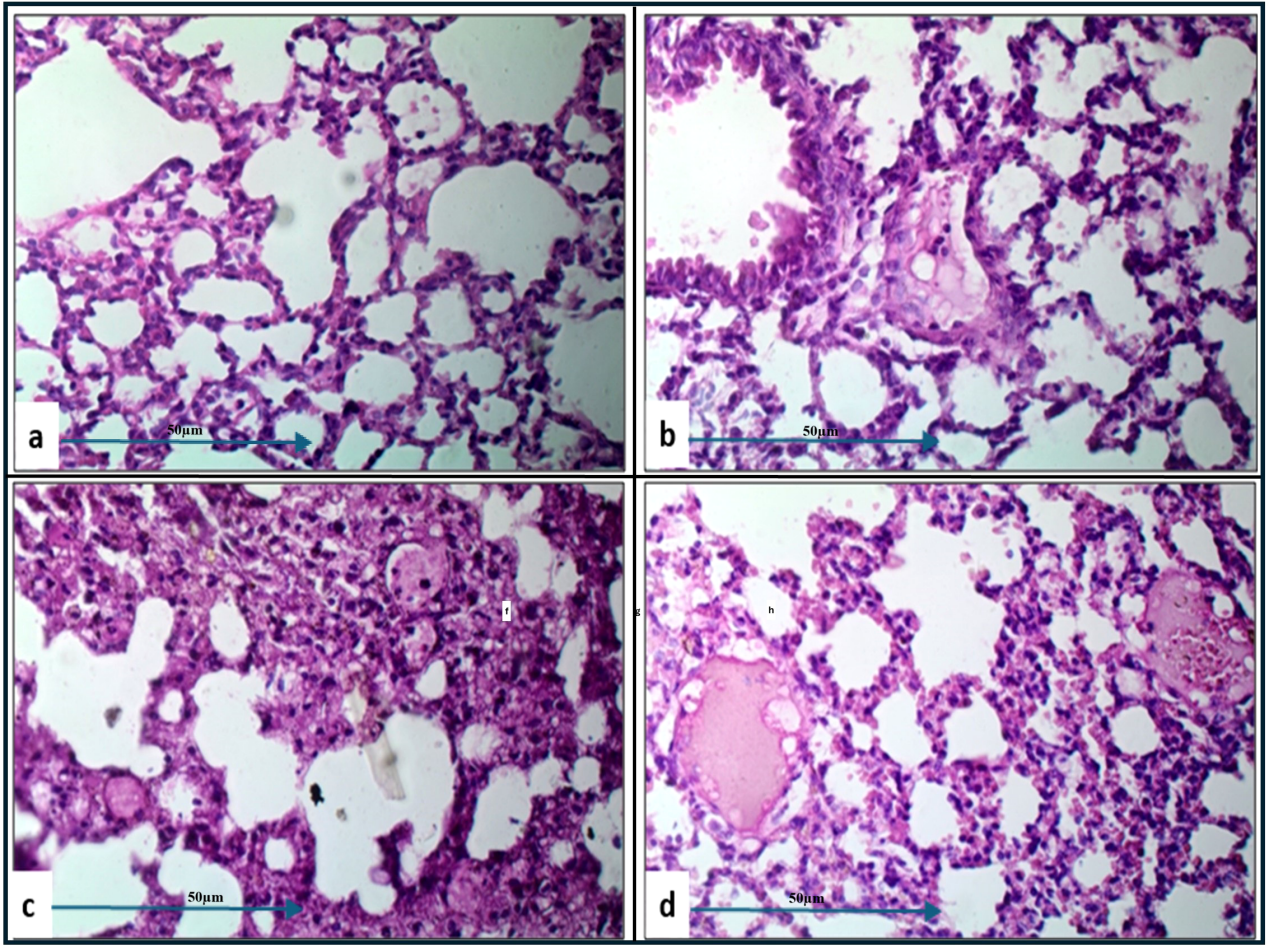
Histological examination of lung tissue from challenged groups treated with combination therapies (H&E, 400x magnification). Histopathological analysis of these groups, challenged with *A. baumannii* **(a)**, *E. coli* **(b)**, *K. pneumoniae* **(c)**, and *P. aeruginosa* **(d)**, showed significant improvement in alveolar wall collapse and congestion in **(a, b)**, with moderate improvement in **(d)**. Alveolar emphysema was pronounced in **(a, b, d)**, and moderate in **(c)**. Marked alveolar edema associated with hemorrhage was observed in **(c)**. Pulmonary vasculature changes, including thrombus formation and neutrophil infiltration, were detected in all groups.

Histopathological examination of lung tissue from unchallenged untreated groups (**Figure 3A**) compared to the challenged untreated groups infected with *A. baumannii* (**Figure 4a**), *E. coli* (**Figure 4b**), *K. pneumoniae* (**Figure 4c**), and *P. aeruginosa* (**Figure 4d**) revealed significant pathological changes. These included collapse of alveolar walls, emphysema, congestion, hemorrhage/edema, as well as pulmonary vascular alterations such as neutrophil infiltration and thrombus formation, with the highest severity scores observed. In the group infected with *A. baumannii*, amikacin alone, as shown in **Figure 5a**, led to moderate alveolar wall congestion (++), whereas the combination therapy, as illustrated in **Figure 6a**, resulted in a mild severity (+), suggesting a potential ameliorative effect of the plant extract. Similarly, interstitial neutrophil infiltration was moderately severe with amikacin treatment (++), but the combination therapy reduced this to a mild response (+). Alveolar edema and hemorrhage followed a comparable pattern, with amikacin showing moderate severity (++) and the combination therapy reducing this to a mild level (+), indicating a reduction in overall lesion severity. Damage to pneumocytes I and endothelial cells was moderately severe with amikacin (++), but notably absent (-) with the combination treatment, suggesting protective benefits of *J. candicans* extract against cellular damage. Severe thrombosis (+++) within the alveolar vasculature was observed with amikacin alone, but this was significantly reduced to mild (+) with the combined treatment. Furthermore, alveoli filled with granular necrotic debris and minimal cellular reaction were moderately severe (++) with amikacin, but this decreased to a mild severity (+) with the combination treatment, and a similar trend was noted in alveolar collapse, with scores reduced from moderate (++) to mild (+) in the combination group (**Table 4**).

**Table 4:** Severity levels of lung and spleen in challenged mice treated with amikacin alone or in combination with *J. candicans* extract.

	*A. baumnii*		*E. coli*		*K. pneumoniae*		*P. aeruginosa*	
	Amikacin	Plant/Amikacin	Amikacin	Plant/Amikacin	Amikacin	Plant/Amikacin	Amikacin	Plant/Amikacin
Pulmonary Sepsis Score Lesion Diffuse Alveolar Damage (DAD)								
Alveolar wall congestion	**++**	**+**	**+++**	**+**	**++**	**++**	**++**	**++**
interstitial neutrophil infiltration	**++**	**+**	**+++**	**+**	**+++**	**+**	**++**	**++**
Alveolar: Edema/hemorrhage	**++**	**+**	**+++**	**+**	**+++**	**+++**	**++**	**+**
Pneumocytes I& endothelial cells damage	**++**	**-**	**+++**	**-**	**++**	**+**	**++**	**-**
Alveolar vasculatures: - Thrombosis	**+++**	**+**	**+++**	**+**	**++**	**++**	**+++**	**++**
Alveoli filled with granular necrotic debris and minimal cellular reaction	**++**	**+**	**+++**	**+**	**++**	**++**	**+**	**+**
Alveolar collapse	**++**	**+**	**+++**	**+**	**++**	**++**	**++**	**++**
Splenic Score Lesion								
Apoptosis/necrosis of Lymphocytes	**+**	**-**	**++**	**+**	**-**	**-**	**+++**	**+**
Fibrosis and/or hyaline deposition	**-**	**-**	**-**	**-**	**-**	**++**	**+**	**+**

The symbols –, +, ++, and +++ represent the severity of histopathological lesions observed in lung and spleen tissues. A minus (–) indicates absence of the lesion, (+) denotes mild severity, (++) indicates moderate severity, and (+++) reflects severe or extensive tissue damage.

For mice challenged with *E. coli*, the lesions were generally more severe, with amikacin treatment alone (**Figure 5B**) resulting in marked alveolar wall congestion (+++), extensive interstitial neutrophil infiltration (+++), and severe pneumocyte and endothelial cell damage (+++). However, the combination of amikacin and *J. candicans* extract (**Figure 6b**) substantially mitigated these effects, reducing alveolar wall congestion and interstitial neutrophils and pneumocyte damage to mild (+). This reduction in severity extended to alveolar vasculature thrombosis, where the combination treatment decreased lesion scores from severe (+++) to mild (++), and alveolar collapse from (+++) to (+), indicating a significant reduction in overall damage when using the combined treatment (**Table 4**).

For *K. pneumoniae*-challenged mice, treatment with amikacin alone (**Figure 5c**) resulted in interstitial neutrophil infiltration with uniformly severe lesions (+++). However, the combination treatment (**Figure 6c**) significantly reduced these scores to mild levels. Pneumocyte damage, which was moderate in the amikacin-treated group, was further reduced to mild levels in the combination therapy group. Despite these improvements, alveolar edema and hemorrhage exhibited severe lesions (+++) with both treatments. Additionally, the presence of alveoli filled with granular necrotic debris, as well as alveolar wall congestion, thrombosis, and collapse, remained consistently moderate (++) for both treatment options, indicating a persistent level of damage (**Table 4**).

In mice challenged with *P. aeruginosa*, amikacin treatment alone (**Figure 5d**) led to moderate lesions across various parameters, including alveolar wall congestion, interstitial neutrophil infiltration, alveolar collapse, and pneumocyte damage, all with moderate scores (++). The combination therapy with *J. candidus* extract (**Figure 6d**) did not markedly alter these outcomes, as most parameters remained at similar moderate levels (++). The severity of alveoli filled with granular necrotic debris was mild (+) in both treatment groups, showing minimal variation. However, a notable improvement was observed in pneumocyte damage with the combination treatment, which was completely resolved, resulting in a healing score (-). Additionally, the combination therapy reduced alveolar thrombosis severity from severe (+++) in the amikacin treated group to moderate (++). Alveolar edema and hemorrhage also improved, from moderate lesions with amikacin treatment to mild lesions with the combination therapy. These findings suggest that while the combination therapy significantly enhanced pneumocyte healing and reduced Alveolar edema, hemorrhage, and thrombosis severity, other aspects such as lung interstitial infiltration, alveolar collapse, pneumocyte damage, and necrotic debris were less responsive to the combined treatment approach (**Table 4**).

**Figure 7:**
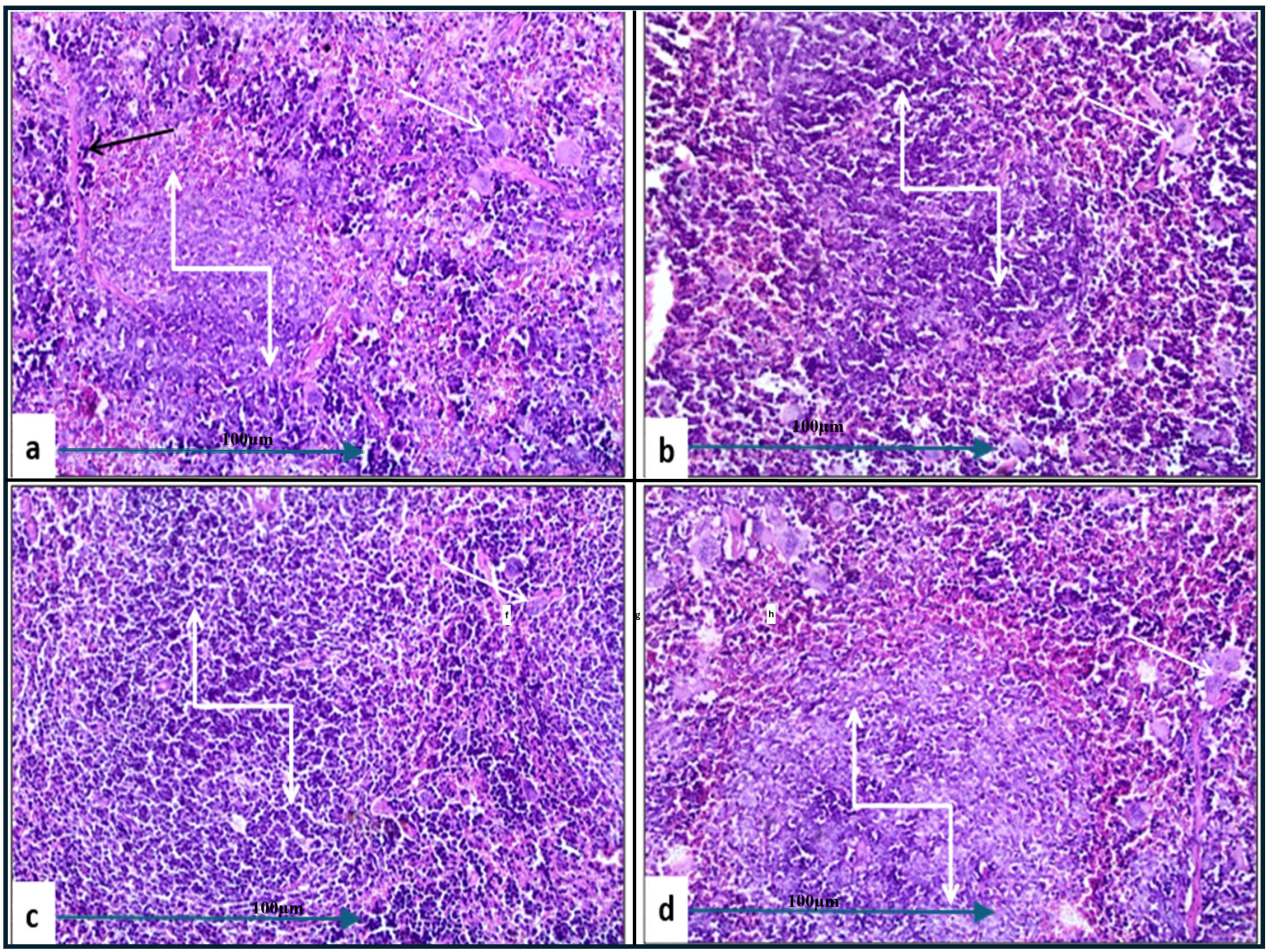
Histological examination of splenic tissue from the control positive (challenged and untreated) group (H&E, 200x magnification). Histopathological analysis of spleen samples from groups challenged with *A. baumannii* **(a)**, *E. coli* **(b)**, *K. pneumoniae* **(c)**, and *P. aeruginosa* **(d)** showed significant alterations in lymphoid follicles (truncated arrows), with lymphoid depletion observed in **(a, c, d)**. Additionally, a high incidence of multinucleated giant cells (white arrows) was noted across all groups. Prominent thick fibrous bands were observed in **(a)** (black arrow).

**Figure 8:**
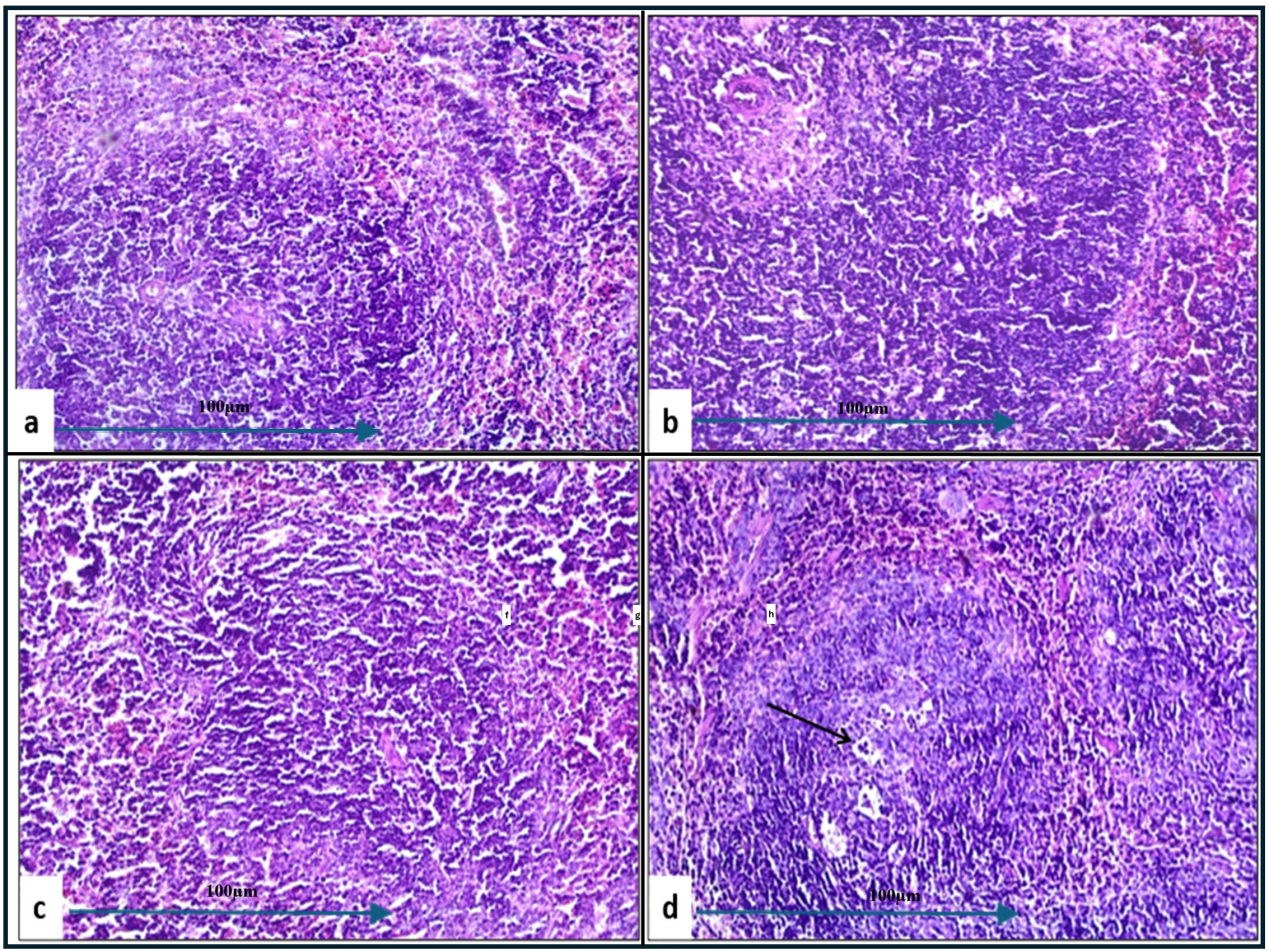
Histological examination of splenic tissue from challenged groups treated with amikacin (H&E, 200x magnification). Histopathological analysis of spleen samples from amikacin-treated groups challenged with *A. baumannii* **(a)**, *E. coli* **(b)**, *K. pneumoniae* **(c)**, and *P. aeruginosa* **(d)** revealed well-developed lymphoid follicles in **(a, b, d)**, while poorly developed follicles were observed in **(c)**. Additionally, lymphocyte degeneration was noted in group **(d)** (black arrow).

**Figure 9:**
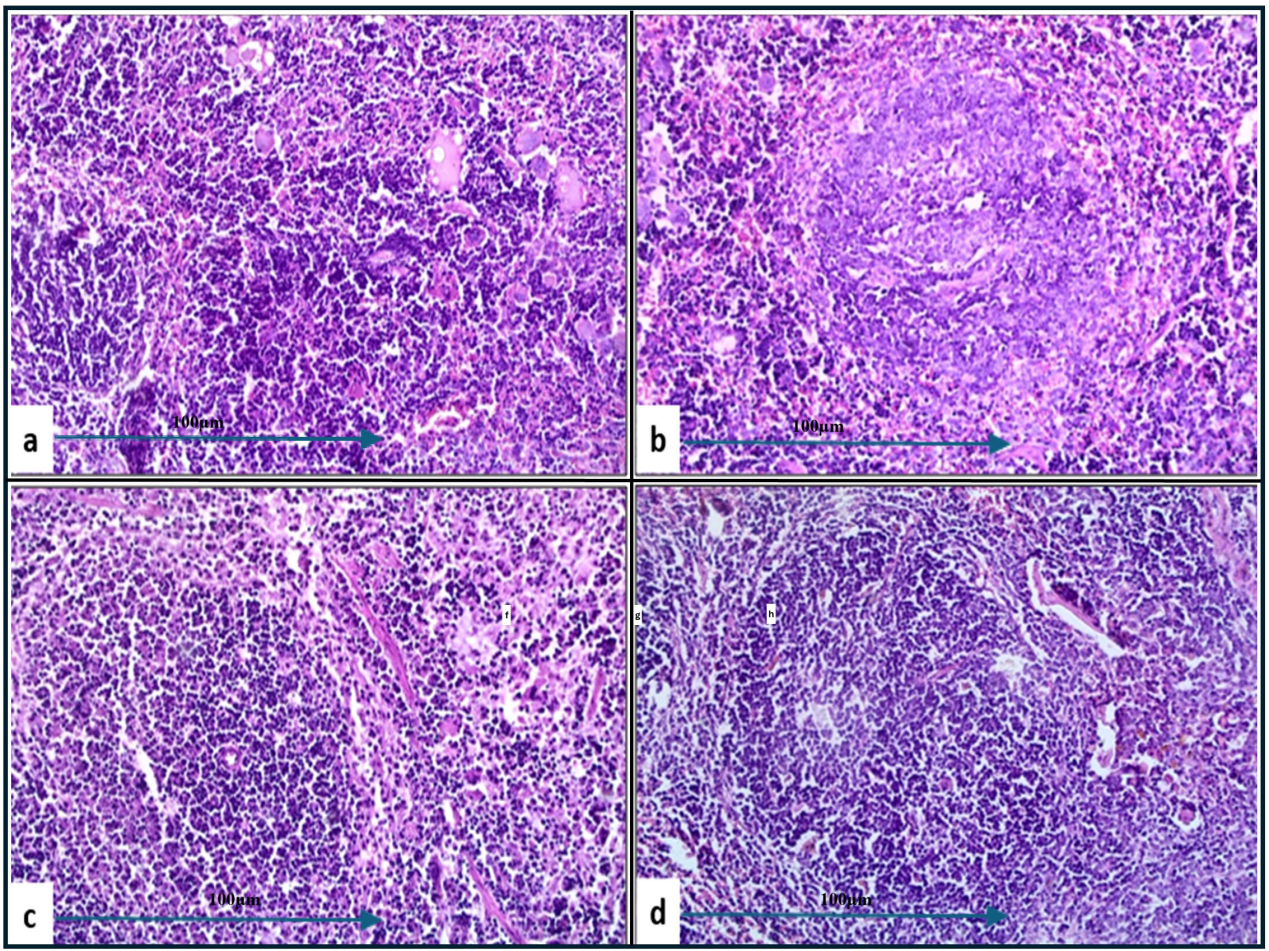
Histological examination of splenic tissue from challenged groups treated with combination therapies (H&E, 200x magnification). Histopathological analysis of spleen samples from these groups, challenged with *A. baumannii* **(a)**, *E. coli* **(b)**, *K. pneumoniae* **(c)**, and *P. aeruginosa* **(d)**, revealed well-developed lymphoid follicles in **(b–d)**, with notable lymphocyte depletion in **(b)**. Additionally, lymphocyte degeneration was observed in **(d)** and **(a)** (black arrow). Well-developed follicles were also seen in group **(a)**.

In the histopathological study comparing splenic tissue from unchallenged untreated mice (control negative) shown in **Figure 3b** with that from untreated challenged mice (**Figure 7**) infected with *A. baumannii* (**Figure 7a**), *E. coli* (**Figure 7b**), *K. pneumoniae* (**Figure 7c**), and *P. aeruginosa* (**Figure 7d**), the challenged mice exhibited a high severity score with significant pathological changes. There was a notable increase in apoptosis and necrosis of lymphocytes, indicating substantial cellular damage and death within the spleen. Furthermore, the presence of considerable fibrosis and/or hyaline deposition in the splenic tissue points to chronic inflammation and tissue remodeling. These results highlight the severe impact of the infections on the spleen, showcasing extensive tissue damage and a compromised immune response. The splenic score lesion analysis of mice treated with amikacin alone (**Figure 8**) or in combination with *J. candicans* extract (**Figure 9**) showed notable differences in the severity of apoptosis/necrosis of lymphocytes and fibrosis/hyaline deposition across the different bacterial infections. For mice challenged with *A. baumannii*, treatment with amikacin (**Figure 8a**) resulted in mild lymphocyte apoptosis/necrosis (+), whereas the combination with *J. candicans* extract (**Figure 9a**) showed no detectable apoptosis/necrosis (-), suggesting a protective effect of the plant extract. Additionally, there was no fibrosis or hyaline deposition observed in either the amikacin alone or the combination treatment groups (-), indicating minimal splenic tissue remodeling in response to the infection or treatment. In the case of *E. coli* infection, amikacin treatment (**Figure 8b**) led to moderate lymphocyte apoptosis/necrosis (++), while the combination with *J. candicans* (**Figure 9b**) significantly reduced this to mild levels (+), further supporting the potential protective role of the plant extract against splenic damage. No fibrosis or hyaline deposition was detected in either treatment group (-), suggesting that the treatments did not induce significant fibrotic changes within the spleen (**Table 4**).

For *K. pneumoniae* infections, neither amikacin alone (**Figure 8c**) nor the combination with *J. candicans* extract (**Figure 9c**) resulted in detectable lymphocyte apoptosis/necrosis or fibrosis/hyaline deposition (-), indicating a lack of observable splenic lesions with these treatments. This suggests that the bacterial strain or the specific conditions did not provoke splenic damage under these treatment regimens. In mice challenged with *P. aeruginosa*, treatment with amikacin alone (**Figure 8d**) resulted in severe lymphocyte apoptosis/necrosis (+++), highlighting a significant degree of splenic damage. However, the combination with *J. candicans* extract (**Figure 9d**) markedly reduced this severity to mild levels (+), demonstrating a substantial protective effect. For fibrosis and hyaline deposition, amikacin alone showed mild changes (+), whereas the combination treatment showed no signs of fibrosis or hyaline deposition (-), further indicating that the extract may help mitigate fibrotic processes in the spleen (**Table 4**).

Overall, the inclusion of *J. candicans* extract with amikacin demonstrated a general trend of reduced lesion severity across several parameters, particularly in cases involving *A. baumannii* and *E. coli*, where notable reductions were observed in alveolar congestion, neutrophil infiltration, and cellular damage. This combination therapy appeared to enhance the therapeutic effects of amikacin, reducing the severity of pulmonary sepsis. However, its efficacy varied depending on the specific pathogen, as seen with *K. pneumoniae* and *P. aeruginosa*, where the combination showed limited or no additional benefit in terms of reducing lesion severity. In the spleen, the combination therapy consistently led to reduced severity of lymphocyte apoptosis/necrosis and prevented fibrosis or hyaline deposition in most bacterial challenges, especially with *A. baumannii*, *E. coli*, and *P. aeruginosa*. These findings highlight the potential of *J. candicans* extract to enhance the protective effects of amikacin, offering additional support in treating bacterial infections associated with significant alveolar, vascular, and splenic damage, although its effectiveness may depend on the specific pathogen involved.

## Discussion

Bloodstream infections (BSIs), specifically those caused by MDR Gram-negative bacterial pathogens, are associated with high morbidity and mortality rates due to the difficulty of treating with the available antimicrobial agents (Tawfik et al., 2023). In addition, it is considered as a major public health concern, particularly in Egypt. Sepsis-related septic shock may arise from a complex pathological reaction such as the hyperinflammatory host response to bacterial infection (Usmani et al., 2021). This study aimed to explore the prevalence and antimicrobial resistance patterns of Gram-negative bacteremia and to assess the potential of medicinal plant extracts as adjunctive treatments. In this study, the prevalence rate of 58.25% for Gram-negative pathogens in blood cultures among septic patients aligns with previous studies that report similar or higher rates of Gram-negative bacteremia in septic patients (Kumar et al., 2016; Tumbarello et al., 2019). *E. coli* and *K. pneumoniae* were the predominant pathogens, which is consistent with global trends where these organisms are frequently implicated in hospital-acquired infections (Bert et al., 2020). The high prevalence of MDR (79.4%) observed in this study, particularly among *P. aeruginosa* and *A. baumannii*, corroborates findings from other regions where these pathogens have shown alarming resistance patterns (Giske et al., 2018; Vardakas et al., 2019). The resistance of Gram-negative isolates to multiple antibiotics, including aztreonam, ampicillin-sulbactam, and various beta-lactams, reflects a broad decline in the efficacy of these agents. This observation is in line with recent reports of increasing resistance rates that complicate the management of Gram-negative infections (Nordmann et al., 2019). The consistent susceptibility of isolates to colistin, a last-resort antibiotic, is notable, though its use is often limited by toxicity and availability (Landman et al., 2008).

Medicinal plant extracts have been documented to possess a range of biological activities, including antimicrobial, anti-inflammatory, and antioxidant properties (Mehta et al., 2001; Vaou et al., 2021a). Our findings showed that the tested multidrug-resistant (MDR) bacterial strains exhibited varying degrees of sensitivity to all the desert-adapted plant extracts examined. The *J. candicans* was the most effective against MDR Gram-negative bacteria, with significant inhibition zones and low minimum inhibitory concentration (MIC) values. This supports previous research indicating that *J. candicans* possesses potent antimicrobial properties (Nogueira et al., 2013). These results may be attributed to the high content of quercetin and kaempferol related to flavonoids as was previously reported (Valero et al., 2013). In addition, the essential oils of *J. candicans* may be also responsible for antimicrobial activity against *B. subtilis* and *P. aeruginosa* (Hammerschmidt et al., 1993; El Yaagoubi et al., 2021). The antimicrobial efficacy of *J. candicans*, while promising, is limited by practical challenges such as achieving the necessary MIC levels due to issues with bioavailability and potential toxicity. To achieve the MIC values, it implies that substantial concentrations of the extract are required to effectively inhibit bacterial growth, which may not be feasible in clinical settings due to difficulties in maintaining these levels in the bloodstream and potential adverse effects (Nogueira et al., 2013). Therefore, *J. candicans* is more effectively used in combination with conventional antibiotics, where it can enhance the overall therapeutic efficacy and potentially reduce the dosage and side effects of both agents (Sawai et al., 2004; Ahmad and Mehmood, 2020). The synergistic effect of combining *J. candicans* with amikacin, resulting in reduced MIC values and conversion of resistant strains to sensitive phenotypes, highlights the potential of this combination therapy. This synergistic interaction is consistent with previous studies that have highlighted the potential of combining plant extracts with conventional antibiotics to enhance therapeutic efficacy and overcome resistance (Sawai et al., 2004; Ahmad and Mehmood, 2020). Such findings suggest that integrating *J. candicans* into treatment regimens could offer a viable strategy for managing infections that are otherwise challenging to treat with standard antibiotics alone.

Despite the critical importance of eradicating Gram-negative bacteria in septicemia, the host’s inflammatory response is recognized as a more significant cause of mortality in septic patients. Monitoring organ dysfunction related to the immune response and inflammatory reactions is crucial in managing sepsis (Nong et al., 2023). Consequently, the anti-inflammatory potential of the desert-adapted plant extracts in our study was evaluated as a key factor in sepsis treatment. Inhibiting COX-2 may facilitate the treatment of multidrug-resistant (MDR) infections through several interconnected mechanisms. COX-2 is an enzyme that plays a significant role in the inflammatory process by catalyzing the conversion of arachidonic acid to prostaglandins, which are key mediators of inflammation. During an infection, especially one caused by MDR pathogens, the inflammatory response can be exaggerated, leading to tissue damage and worsening clinical outcomes (Nathan, 2002). However, combining antibiotics with non-steroidal anti-inflammatory drugs (NSAIDs) can pose several potential disadvantages, including an increased risk of gastrointestinal irritation, bleeding, renal impairment, and liver toxicity due to the side effects of both drug classes. Drug interactions may occur, as some antibiotics can alter NSAID metabolism, leading to enhanced toxicity or reduced efficacy. Additionally, NSAIDs can suppress certain aspects of the immune response, hindering the body’s ability to fight infections, and may complicate treatment regimen due to the need for careful monitoring. There is also a risk of masking important clinical symptoms, such as fever and pain, which could impede accurate diagnosis and treatment assessment. Therefore, healthcare providers must evaluate the necessity of this combination therapy on a case-by-case basis (Adams and Lichtenstein, 2017). Therefore, it is essential to explore alternative natural COX-2 inhibitors to mitigate or eliminate these concerns.

Our findings demonstrated that all four tested plant extracts achieved a COX-2 selective index comparable to celecoxib (ranging from 0.23 to 0.28), indicating preferential inhibition of COX-2 and highlighting their value as anti-inflammatory agents. Additionally, oxidative stress plays a pivotal role in the pathophysiology of septic shock, contributing to multiple organ failure, ischemia-reperfusion injury, and acute respiratory distress syndrome (Aisa-Alvarez et al., 2020). Antioxidants may modulate inflammation in septic shock patients and could be integrated into standard therapeutic protocols. To assess the antioxidant potential of the tested plant extracts, we utilized the DPPH scavenging assay, a reliable method for measuring the total antioxidant capacity of herbal extracts (Huang et al., 2005). Notably, our study revealed that *J. candicans* extract exhibited the highest DPPH free radical scavenging activity, with the lowest IC_50_ value. Such properties are vital for combating oxidative stress-related conditions and enhancing overall health benefits (Miliauskas et al., 2004; Pires et al., 2015). Therefore, *J. candicans* plays a crucial role in therapeutic applications due to its combined antioxidant and anti-inflammatory properties, alongside its antimicrobial activity. Concurrently, its antimicrobial activity targets and inhibits pathogenic bacteria, when used in combination therapies, making it a valuable candidate for treating infections while managing inflammation and oxidative damage (Nogueira et al., 2013; Jang et al., 2020).

Histopathological analysis demonstrated that *J. candicans*, when used in conjunction with amikacin at a sub-inhibitory concentration (0.5 MIC), notably reduced the severity of pulmonary and splenic damage in mice models. This protective effect was evident in reduced alveolar congestion, decreased interstitial neutrophil infiltration, and less severe tissue damage compared to treatment with amikacin alone. Such findings underscore the potential of *J. candicans* to enhance the efficacy of conventional antibiotics and provide a protective effect against bacterial-induced tissue damage (Gupta et al., 2018; Kumari et al., 2020). Previous studies have supported the notion that plant extracts, including *J. candicans*, can mitigate the severity of inflammatory and infectious lesions. For instance, research has shown that various plant-derived compounds possess anti-inflammatory properties that reduce tissue damage and improve recovery outcomes in infectious models (Gupta et al., 2018). Additionally, another study highlighted the ability of plant extracts to alleviate inflammatory responses and oxidative stress, contributing to tissue protection during infections (Kumari et al., 2020). However, the limited efficacy observed with specific pathogens points to the need for continued investigation. The variability in response underscores the importance of tailoring treatment strategies to specific pathogens and optimizing the use of plant extracts in combination with antibiotics (Gupta et al., 2018; Kumari et al., 2020; Sharma et al., 2019).

Finally, we propose that the natural extracts investigated in our study may serve as potential adjunct therapies to enhance the effectiveness of standard antimicrobial treatments, particularly in cases where traditional antibiotics are ineffective due to the rise of MDR pathogens. The concept of adjunct therapy involves using these extracts in conjunction with conventional antibiotics to achieve a synergistic effect, thereby increasing the overall efficacy of treatment. For instance, certain extracts may possess properties that can disrupt biofilms formed by MDR bacteria, improve the host immune response, or possess inherent antimicrobial activity that complements the action of antibiotics. In clinical settings, where infections caused by MDR pathogens present significant treatment challenges, incorporating these natural extracts could potentially reduce the dosage and duration of antibiotic therapy required, thereby minimizing side effects and the risk of further resistance development. Additionally, using these extracts may help to mitigate the adverse effects commonly associated with antibiotics, such as disruption of the gut microbiota. To support the transition from laboratory research to practical clinical application, it is essential to conduct rigorous clinical trials that evaluate the safety, efficacy, and appropriate dosing of these extracts in human subjects. Such studies would help clarify the role of these natural alternatives as adjunct therapies, paving the way for their integration into clinical protocols aimed at combating MDR infections.

## Conclusion

This study revealed a high prevalence of Gram-negative bacteremia with significant multidrug resistance, particularly among *E. coli* and *K. pneumoniae*, posing a major treatment challenge. Notably, *J. candicans* extract demonstrated strong antimicrobial, anti-inflammatory, and antioxidant properties, showing potential as an adjunct to conventional antibiotics like amikacin. The combination of *J. candicans* and amikacin displayed synergistic effects, significantly enhancing antimicrobial efficacy and reducing tissue damage in septic conditions, particularly for *A. baumannii* and *E. coli* infections. These findings underscore the potential of integrating desert-adapted plant extracts into treatment regimens for MDR Gram-negative infections, highlighting the need for alternative therapeutic strategies alongside robust antimicrobial stewardship.

### Strengths of the study

This study presents a novel approach to tackling multidrug-resistant (MDR) bacterial infections by exploring the antimicrobial potential of desert-adapted medicinal plant extracts. The integration of microbiological, biochemical, and histopathological analyses provides a comprehensive evaluation of the therapeutic properties of these extracts. A key strength is the discovery that *J. candicans* extract enhances the efficacy of amikacin, converting resistant bacterial strains into sensitive ones, which holds significant clinical implications. Additionally, the extract exhibits anti-inflammatory (COX-1 and COX-2 inhibition) and antioxidant properties, making it a promising dual-action therapeutic agent. The study also directly addresses the growing crisis of antibiotic resistance in septic patients, an area of high clinical relevance, and demonstrates improved treatment outcomes in an *in vivo* model. The findings suggest that plant-based compounds could serve as adjunct therapies, supporting their potential in future drug development.

### Limitations of the study

Despite its strengths, the study does not investigate the precise molecular mechanisms underlying the antimicrobial and anti-inflammatory effects of the extract, which could provide deeper insights into its mode of action. While the *in vivo* study in mice is a valuable step, larger preclinical and clinical trials are necessary to confirm safety and efficacy in humans. Another limitation is that the long-term impact on bacterial resistance evolution was not assessed, which is critical for determining the sustainability of plant-based adjunct therapies. Lastly, while histopathological analysis indicated reduced tissue damage, detailed toxicity studies are required to ensure the extract’s long-term safety for clinical applications.

## Data Availability

The original contributions presented in the study are included in the article/**Supplementary Material**. Further inquiries can be directed to the corresponding author/s.
