# Supplementary material for: Therapeutic approaches for septicemia induced by multidrug-resistant bacteria using desert-adapted plants
Source: Front Cell Infect Microbiol. 2025 Apr 22;15:1493769. doi: 10.3389/fcimb.2025.1493769 (PMC12052906; doi:10.3389/fcimb.2025.1493769)
Supplement: Supplementary file 1 [file Table1.docx]

| Isolates code  **Table S1: MIC values for the amikacin, *J.candicans, C. tubulosa, T. hirsute, M. ciliata* extract and the FIC values of amikacin */ J.candicans* combination** | Amikacin | *J.candicans* | *C. tubulosa* | *T. hirsuta* | *M. ciliata* | amikacin/  .*J. andicans* | *J.candicans /*  amikacin | FIC amikacin | FIC *J.candicans* | FIC total |
| --- | --- | --- | --- | --- | --- | --- | --- | --- | --- | --- |
| E1 | 128 | 250 | 500 | 500 | 1000 | 16 | 62.5 | 0.125 | 0.25 | 0.375 |
| E2 | 128 | 250 | 500 | 500 | 1000 | 16 | 62.5 | 0.125 | 0.25 | 0.375 |
| E3 | 128 | 250 | 500 | 500 | 1000 | 32 | 62.5 | 0.25 | 0.25 | 0.5 |
| E4 | 128 | 500 | 500 | 500 | 1000 | 8 | 62.5 | 0.0625 | 0.125 | 0.1875 |
| E5 | 256 | 250 | 500 | 500 | 1000 | 16 | 62.5 | 0.0625 | 0.25 | 0.3125 |
| E6 | 128 | 250 | 250 | 500 | 500 | 16 | 31.25 | 0.125 | 0.125 | 0.25 |
| E7 | 256 | 250 | 500 | 500 | 1000 | 32 | 62.5 | 0.125 | 0.25 | 0.375 |
| E8 | 512 | 500 | 500 | 1000 | 1000 | 128 | 125 | 0.25 | 0.25 | 0.5 |
| E9 | 256 | 250 | 500 | 500 | 1000 | 16 | 31.25 | 0.0625 | 0.125 | 0.1875 |
| E10 | 512 | 250 | 500 | 500 | 1000 | 64 | 62.5 | 0.125 | 0.25 | 0.375 |
| E11 | 512 | 250 | 500 | 500 | 1000 | 64 | 62.5 | 0.125 | 0.25 | 0.375 |
| E12 | 256 | 250 | 500 | 250 | 1000 | 16 | 125 | 0.0625 | 0.5 | 0.5625 |
| E13 | 256 | 250 | 500 | 500 | 1000 | 64 | 62.5 | 0.25 | 0.25 | 0.5 |
| E14 | 256 | 500 | 500 | 500 | 1000 | 16 | 125 | 0.0625 | 0.25 | 0.3125 |
| K1 | 512 | 500 | 1000 | 1000 | 1000 | 64 | 62.5 | 0.125 | 0.125 | 0.25 |
| K2 | 512 | 500 | 500 | 1000 | 1000 | 64 | 125 | 0.125 | 0.25 | 0.375 |
| K3 | 256 | 500 | 500 | 1000 | 1000 | 64 | 125 | 0.25 | 0.25 | 0.5 |
| K4 | 256 | 500 | 500 | 1000 | 1000 | 64 | 62.5 | 0.25 | 0.125 | 0.375 |
| K5 | 128 | 1000 | 500 | 1000 | 1000 | 16 | 125 | 0.125 | 0.125 | 0.25 |
| K6 | 512 | 500 | 500 | 1000 | 1000 | 64 | 125 | 0.125 | 0.25 | 0.375 |
| K7 | 256 | 500 | 500 | 1000 | 1000 | 16 | 125 | 0.0625 | 0.25 | 0.3125 |
| K8 | 256 | 250 | 250 | 1000 | 500 | 64 | 32.25 | 0.25 | 0.129 | 0.379 |
| K9 | 512 | 500 | 500 | 1000 | 1000 | 64 | 62.5 | 0.125 | 0.125 | 0.25 |
| K10 | 64 | 500 | 500 | 1000 | 1000 | 8 | 125 | 0.125 | 0.25 | 0.375 |
| K11 | 128 | 500 | 500 | 500 | 1000 | 16 | 125 | 0.125 | 0.25 | 0.375 |
| K12 | 256 | 1000 | 500 | 1000 | 1000 | 64 | 250 | 0.25 | 0.25 | 0.5 |
| K13 | 64 | 250 | 1000 | 1000 | 500 | 16 | 32.25 | 0.25 | 0.129 | 0.379 |
| K14 | 256 | 500 | 500 | 1000 | 1000 | 64 | 125 | 0.25 | 0.25 | 0.5 |
| A1 | 256 | 500 | 1000 | 1000 | 1000 | 16 | 125 | 0.0625 | 0.25 | 0.3125 |
| A2 | 256 | 500 | 1000 | 1000 | 1000 | 64 | 125 | 0.25 | 0.25 | 0.5 |
| A3 | 128 | 500 | 1000 | 1000 | 1000 | 16 | 62.5 | 0.125 | 0.125 | 0.25 |
| A4 | 256 | 500 | 1000 | 1000 | 1000 | 64 | 62.5 | 0.25 | 0.125 | 0.375 |
| A5 | 256 | 500 | 500 | 1000 | 500 | 64 | 62.5 | 0.25 | 0.125 | 0.375 |
| A6 | 128 | 500 | 1000 | 1000 | 1000 | 16 | 125 | 0.125 | 0.25 | 0.375 |
| A7 | 512 | 1000 | 1000 | 1000 | 1000 | 64 | 125 | 0.125 | 0.125 | 0.25 |
| A8 | 512 | 500 | 1000 | 500 | 1000 | 128 | 125 | 0.25 | 0.25 | 0.5 |
| A9 | 128 | 1000 | 1000 | 1000 | 1000 | 16 | 250 | 0.125 | 0.25 | 0.375 |
| P1 | 128 | 500 | 1000 | 1000 | 1000 | 16 | 62.5 | 0.125 | 0.125 | 0.25 |
| P2 | 64 | 500 | 1000 | 1000 | 1000 | 8 | 125 | 0.125 | 0.25 | 0.375 |
| P3 | 512 | 500 | 1000 | 1000 | 1000 | 64 | 125 | 0.125 | 0.25 | 0.375 |

- The isolates highlighted in light blue indicate amikacin-resistant strains that were converted to sensitive phenotypes upon treatment with J. candicans extract.
