# Supplementary figures and images for: Therapeutic approaches for septicemia induced by multidrug-resistant bacteria using desert-adapted plants
Source: Front Cell Infect Microbiol. 2025 Apr 22;15:1493769. doi: 10.3389/fcimb.2025.1493769 (PMC12052906; doi:10.3389/fcimb.2025.1493769)

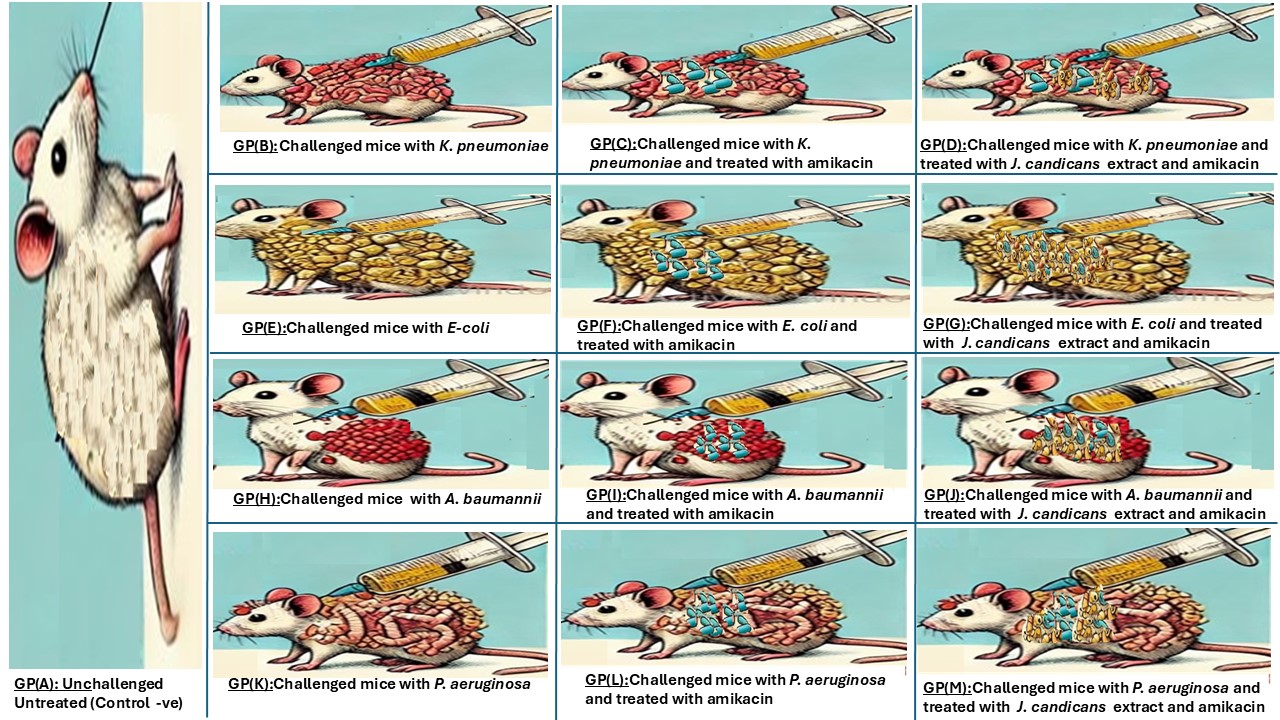

Supplement: Supplementary file 2 [file Image1.jpeg]

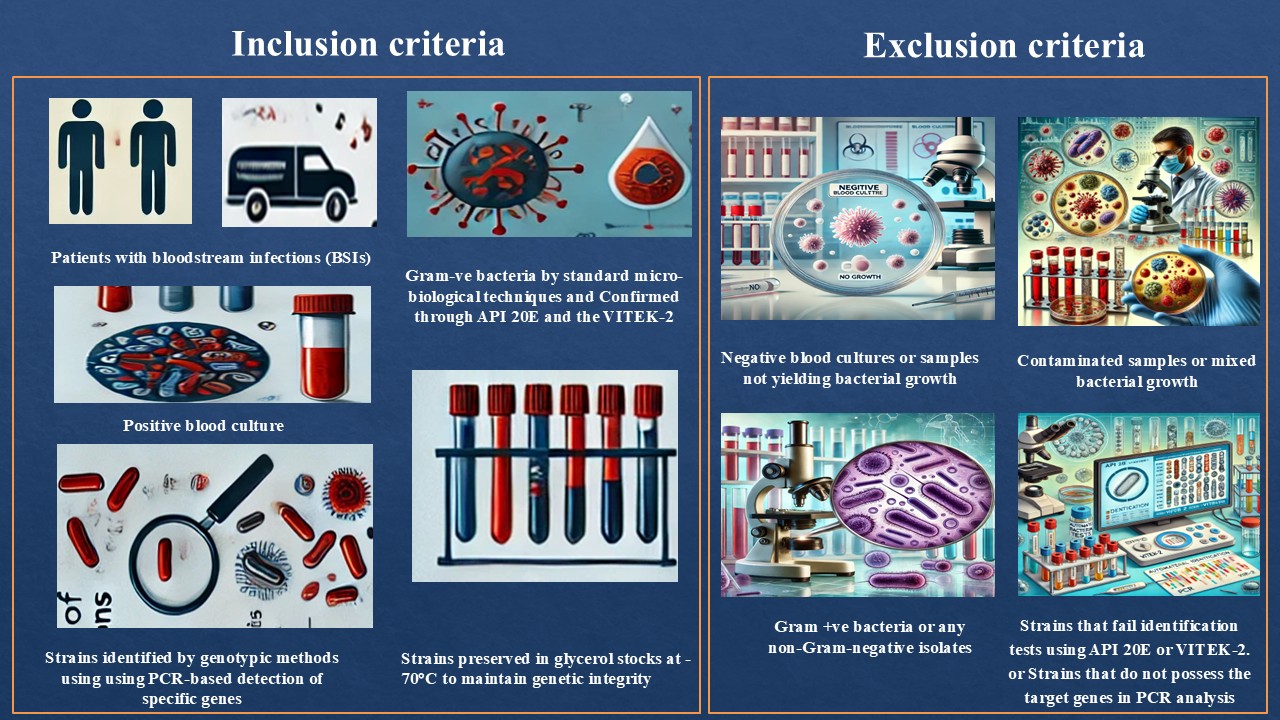

Supplement: Supplementary file 3 [file Image2.jpeg]
